# Extending deterministic transport capabilities for very-high and ultra-high energy electron beams

**DOI:** 10.1038/s41598-023-51143-8

**Published:** 2024-02-02

**Authors:** Ahmed Naceur, Charles Bienvenue, Paul Romano, Cornelia Chilian, Jean-François Carrier

**Affiliations:** 1 École Polytechnique, SLOWPOKE Nuclear Reactor Laboratory, Nuclear Engineering Institute, Montréal, H3T1J4 Canada; 2 École Polytechnique, Engineering Physics Department, Biomedical Engineering Institute, Montréal, H3T1J4 Canada; 3 Computational Science Division, Argonne National Laboratory, Lemont, IL 60439 USA; 4 Department of Physics, Université de Montréal, Montréal, H3T1J4 Canada; 5 CRCHUM, Centre hospitalier de l’Université de Montréal, Montréal, H2L4M1 Canada

**Keywords:** Radiotherapy, Oncology, Engineering, Applied physics, Nuclear physics, Experimental nuclear physics, Statistical physics, Computational science, Scientific data

## Abstract

Focused Very-High Energy Electron (VHEE, 50–300 MeV) and Ultra-High Energy Electron (UHEE, > 300 MeV) beams can accurately target both large and deeply seated human tumors with high sparing properties, while avoiding the spatial requirements and cost of proton and heavy ion facilities. Advanced testing phases are underway at the CLEAR facilities at CERN (Switzerland), NLCTA at Stanford (USA), and SPARC at INFN (Italy), aiming to accelerate the transition to clinical application. Currently, Monte Carlo (MC) transport is the sole paradigm supporting preclinical trials and imminent clinical deployment. In this paper, we propose an alternative: the first extension of the nuclear-reactor deterministic chain Njoy-Dragon for VHEE and UHEE applications. We have extended the Boltzmann-Fokker-Planck (BFP) multigroup formalism and validated it using standard radio-oncology benchmarks, complex assemblies with a wide range of atomic numbers, and comprehensive irradiation of the entire periodic table. We report that $99\%$ of water voxels exhibit a BFP-MC deviation below $2\%$ for electron energies under $\text{1.5 GeV}$. Additionally, we demonstrate that at least $97\%$ of voxels of bone, lung, adipose tissue, muscle, soft tissue, tumor, steel, and aluminum meet the same criterion between $\text{50 MeV}$ and $\text{1.5 GeV}$. For water, the thorax, and the breast intra-operative benchmark, typical average BFP-MC deviations of $0.3\%$ and $0.4\%$ were observed at $\text{300 MeV}$ and $\text{1 GeV}$, respectively. By irradiating the entire periodic table, we observed similar performance between lithium ($Z=3$) and cerium ($Z=58$). Deficiencies observed between praseodymium ($Z=59$) and einsteinium ($Z=99$) have been reported, analyzed, and quantified, offering critical insights for the ongoing development of the Evaluated Nuclear Data File mode in Njoy.

## Introduction

More than half of the 19 million cancer patients, diagnosed worldwide each year, receive radiotherapy (RT) treatment during the course of their disease^1,2^. Survival rates differ starkly between cancer types and conventional RT (CONV-RT) techniques^3,4^. Schulz and Kagan^5^ outlined that CONV-RT 5-year survival rate for endometrium, breast, bladder and colorectal cancers is $87.9\%$, $74.6\%$, $72.6\%$ and $49.8\%$, respectively, while it is only $2.5\%$, $3.8\%$, $4.6\%$ and $10\%$ for pancreas, liver, esophagus and lung cancers.

Cure and mortality patterns depend on Holthusen’s therapeutic window (TW) width^6^; i.e., the difference between the tumor control probability (TCP) and the normal tissue complication probability (NTCP)^7,8^. Oncologists acknowledge that RT survival rate can be improved if (1) current tumor control is guaranteed^9^; (2) the risk of developing fatal secondary malignancies—draining regional lymphatics and lymph nodes—is minimized or preserved^10^; (3) with a marked reduction in the collateral damage inflicted on healthy tissues^11^.

Two ingredients must come together to target a TW’s widening; (1) a technique that ensures dose conformity to the Planning Target Volume (PTV)^12^; and (2) an accurate delivery (and calculation) of radiation dose through heterogeneities^13^. Studies^14–19^ have shown that higher survival rates and lower distant metastases occur when local-regional disease is controlled. Over the years, Volumetric Arc Therapy (VMAT)^20^, Intensity-Modulated Radiation Therapy (IMRT)^21^, 3-Dimensional Conformal Radiotherapy (3DCRT)^22^, Stereotactic Radiosurgery (SRS)^10^, intra-operative radiation therapy (IORT)^23^, modulated electron radiation therapy (MERT)^24^, electron arc therapy (EAT)^25^, dynamic electron arc radiotherapy (DEAR)^26^ and brachytherapy have improved the tumoricidal dose lethality^27^. On the other hand, their capacity to reduce toxicity^28,29^ and critical normal-tissue complications^30–32^ is strongly questioned and debated^33^. This is because engineering and computing improvements^34^ are limited by the ballistic properties of the incident particle^35,36^. This has resulted in the current state-of-the-art clinical dose fractionation and escalation practices (e.g., $\text{2.0 Gy}$/fraction, 4–6 fractions/week, 25–39 sessions/treatment)^37,38^ for CONV photon RT (CONV-PRT). Compared to photon beams, clinically-used electron beams have a significant advantage, i.e., the sharp decline in dose beyond the maximum^39^. This reduces complications, non-malignant tissue toxicity and widens the TW^40^. However, the electronic buildup profile has limited, over the years, the use of CONV electron RT (CONV-ERT) in the 4–20 MeV range to superficial malignancies (e.g., epithelial or nonmelanoma skin tumor^41^), inoperable and recurrent salivary glands cancer^42^, uveal malignant melanoma^43^, prophylactic breast^44^ and recently for intraoperative genitor-urinary malignancies, e.g., cervical, bladder, renal, endometrial and prostate cancers^45^. Ronga et al.^4^ explain that, unlike IMRT and VMAT, no effort has been made to implement complex intensity-modulated ERT. A higher tumoricidal dose conformity with the possibility of reaching deep-seated tumors—with substantial sparing properties—was only possible with proton^46^ or $^{12}$C^47^ ion Bragg peak. Covering the entire targeted tumor volume is possible with the superposition and modulation of the incident hadronic beam energy^48–53^. This results in a spread-out Bragg peak (SOBP) which has the disadvantage of being very sensitive to tissue density inhomogeneity^54–57^.

Setting up these beams kept requiring space-intensive infrastructure and highly-expensive accelerators and hadron transport systems^58^. The proton beams provide potential cost-efficiency, exclusively, for pediatric brain neoplasms^59^, selectively identified breast carcinomas with elevated cardiotoxicity risk^60^, locoregionally advanced non-small cell lung cancer (NSCLC)^61,62^, and high-risk head and neck malignancies^63^. It is worth questioning if there is a technique that is as accurate as hadronic beams, as easily clinically deployable as CONV-RT, and less toxic than the latter.

Using Penelope, Desrosiers et al.^64^ and Papiez et al.^65^ were the first to demonstrate the Very-High Energy Electron (VHEE) ballistic capability to reach the most deep-seated human tumor. The authors related the beam’s geometric dimensions to its lateral spread, penumbra and penetration degree, highlighting the potential of an electromagnetic scanned intensity modulation modality. Møller and Mott’s double differential scattering cross sections (DDSC) are inversely proportional to the energy of the incident electron squared. Therefore, lateral scattering is correspondingly reduced with energy increase. Consequently, the VHEE beam’s penumbra is sharper for shallower depths and increases for deep-seated targets. Similarly, the same holds true for Ultra-High Energy Electron (UHEE) beams^66^. In a later work, Desrosiers^67^ reported that the VHEE beam’s dose is minimally affected by surface obliquity or depth heterogeneity, maintaining a sustained dose uniformity at organ-tissue interfaces for various densities of lung, muscle, bone, fat, and air cavities. Subsequently, this was confirmed experimentally by Lagzda et al.^68^ at CERN in Switzerland. Moreover, the experimental dose longitudinal profile was compared to Topas-Geant-4 predictions for a 156 MeV beam, with a small sensitivity of 5–8% confirmed when water density was increased from $\text{0.001 g cm}^{-3}$ to $\text{2.2 g cm}^{-3}$. Glinec et al.^69^ successfully demonstrated the experimental feasibility of producing a $\text{170 MeV}$ compact well-collimated quasi-monoenergetic laser-accelerated beam with a magnetically-focused sharp and narrow transverse penumbra. Notably, Fuchs et al.^70^ showed experimentally that laser-accelerated VHEE beams (150, 185, $\text{250 MeV}$) enhance the quality of a prostate treatment planning. Compared to a clinically approved $\text{6 MV}$ IMRT plan, VHEE penumbra ensured better protection for the rectum and bladder, while the femoral heads systematically received higher doses. Later, in an interesting development, Lagzda^71^ successfully reduced the width of the VHEE beam to $\text{3.0 mm}$ along one axis using two electromagnetic quadrupole triplets and external magnetic fields at the CLEAR facility. Furthermore, Kokurewicz et al.^66,72^ showed that focused beams with energies of 158–$\text{201 MeV}$ can shape, scan, and concentrate the dose into small, well-defined and deep-seated volumetric voxels. In addition, McManus et al.^73^ established the foundation for a new standard clinical dosimetry protocol for FLASH-VHEE beams. Meanwhile, Labate et al.^74^ demonstrated that current laser-plasma accelerators are suitable for preclinical VHEE-RT in-vitro and in-vivo studies. Whitmore^75^ innovatively created an adjusted sum of concentrated VHEE to produce a tumor spread-out electron peak (SOEP). These dose profiles: (1) were doubly, laterally and transversally, shaped; and (2) showed better entrance and lower heterogeneity sensitivity than proton-based SOBP and photon beams. By measuring the neutron yield from a $\text{200 MeV}$ and $\text{2 GeV}$ electron beams, Masilela et al.^76^ concluded that clinical deployment of both VHEE-RT and UHEE-RT would not require additional radioprotection safeguards compared to CONV-RT. Simultaneously, Delorme et al.^77^ found that VHEE-RT has the potential to be more biologically effective than CONV-RT from a macrodosimetric perspective. Furthermore, Svendsen et al.^78^ demonstrated the clinical spatial constraint feasibility of a fractionated stereotactic RT with a focused-laser wakefield VHEE beam. Bohlen ^79^ clinically characterized VHEEs, meticulously exploring the relationships between their ranges, penumbra, energy, field size, and source-axis distance.

As it stands today, with the advancements in radiofrequency (RF) technology for linear colliders^80^, compact accelerating infrastructures—exceeding the $\text{100 MV m}^{-1}$ gradient—are undergoing physical and preclinical VHEE-RT experimentation. Three facilities are currently in use for these purposes; (1) the CERN 220 MeV Linear Electron Accelerator for Research (CLEAR) in Switzerland^81^; (2) the Stanford 120 MeV Next Linear Collider Test Accelerator (NLCTA) in USA^82^; and (3) the INFN 170 MeV Sources for Plasmas Accelerators and Radiation Compton with Laser and Beams (SPARC) in Italy^83^. In addition, at least five other VHEE facilities are under development worldwide, namely (1) the Compact Linear Accelerator for Research and Applications (CLARA) in UK^84,85^; (2) the Photo Injector Test Facility (PITZ) in Germany^86^; (3) the Argonne Wakefield Accelerator (AWA) in USA^87^; (4) the Inverse Compton Scattering Source at the University of Tsinghua (ICSS) in China^88^; and (5) the Stanford Pluridirectional High-energy Agile Scanning Electronic Radiotherapy clinical system (PHASER) in USA^89,90^ for the image-guided FLASH VHEE-RT.

Every study previously mentioned has exclusively used Monte Carlo (MC) codes, currently the main support for VHEE transport. In 2006, the Los Alamos discrete-ordinates Attila $S_N$ solver was introduced^91^ and has since been validated^92,93^ and certified^94–103^ for use in CONV-PRT. In 2023, we proposed an open source nuclear data processing chain, Njoy-Dragon-5, for CONV-ERT (1–20 MeV)^104^. The purpose of this paper is to extend the clinical interest in Njoy-Dragon-5 chain for VHEE (50–300 MeV) and UHEE (> 300 MeV) beams. More precisely, our study aims to: (1) revisit and extend the multigroup state-of-the-art formalism in Njoy; (2) expand the MATXS-formatted^105,106^ electroatomic libraries for VHEE and UHEE beams; (3) evaluate the chain performance using typical VHEE- and UHEE-RT benchmarks; and (4) identify the limitations of the proposed solution at VHEE and UHEE.

## Methods

### Introduction to NJOY workflow

**Figure 1:**
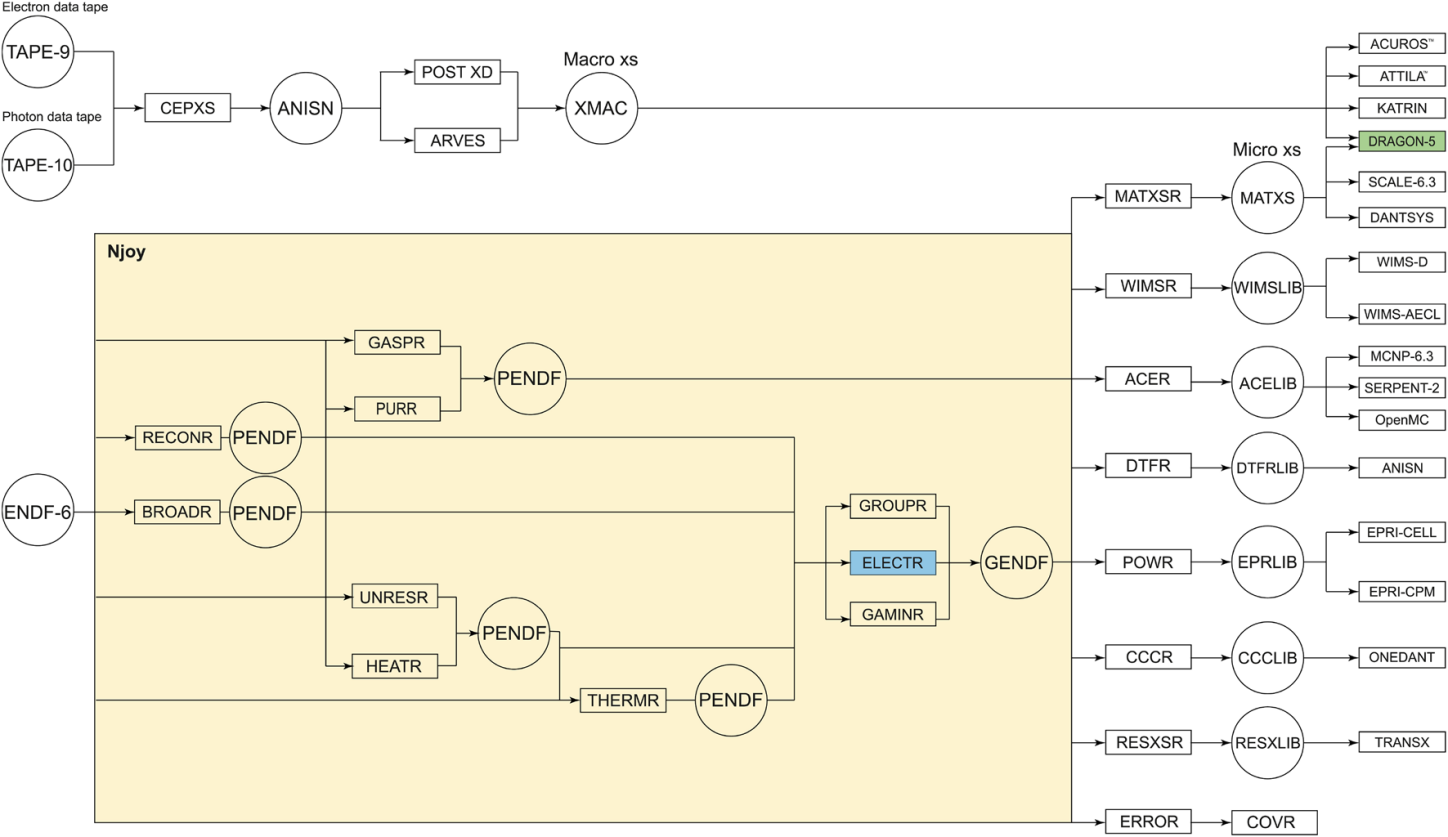
Njoy nuclear data processing system simplified workflow. RECONR resconstructs PENDF cross sections from ENDF; BROADR computes Doppler-broadened cross sections; UNRESR computes self-shielded cross sections in the unresolved range; HEATR produces heat KERMA and radiation damage cross sections; THERMR computes free and bound scatters thermal cross sections; GROUPR applies multigroup theory for neutron cross sections; GAMINR applies multigroup theory for photon cross sections; ELECTR applies multigroup theory for electron cross sections; PURR prepares unresolved-region probability tables for MC neutron transport; GASPR generates gas-production cross sections; ERROR computes multigroup covariance matrices; COVR performs covariance plotting; MATXSR, WIMSR, ACER, DTFR, POWR, CCCR and RESXSR format multigroup and pointwise data for specialized codes and applications.

Proposed in 1973 as a natural successor to the Multigroup Interpretation of Nuclear X-sections (MINX)^107^, the development of Njoy was entirely funded by the U.S. Fast Breeder Reactor^108^ and Weapons Programs^106^. Njoy benefited from the merging of several algorithms and codes, namely ETOPL kernels for Pointwise Evaluated Nuclear Data Files (PENDF) libraries^109^, RESEND for union grid with resonance reconstruction^110^, SIGMA for Doppler-broadening^111^, ETOX for unresolved resonance self-shielding^112^, LAPHANO0 for photonic production^113^, CAMLEG for photonic interaction^114^, FLANGE-II^115^ and HEXSCAT^116^ for thermal neutron scattering, TRANSX for library formatting^105^, SAMMY for Reich-Moore-Limited resonance representation^117^ and MACK for heat production and radiation damage energy production^118^. In 1977 and 1979, Njoy garnered support from the U.S. Electric Power Research Institute (EPRI) and the U.S. Magnetic Fusion Energy Program for the development of Groupwise Evaluated Nuclear Data Files (GENDF) libraries, enabling data formatting compatible with EPRI neutronic solvers and covariance production, respectively. Between 1981 and 2016, Njoy underwent numerous improvements, building upon previous work and leveraging the accumulated experience of numerous international contributors. This collaborative effort resulted in enhanced stability, code maturity, an open-source release in 2016, and a versatile capability for processing various types of data and formats. These include the US. ENDF/B^119,120^, the JEFF libraries in Europe^121^, JENDL in Japan^122^, TENDL^123^ , CENDL in China^124^, BROND^125^ and RUSFOND^126^ in Russia, and the specialized libraries of the International Atomic Energy Agency (IAEA) Nuclear Data Section (NDS)^127–129^. Njoy has earned its distinctiveness and near-domination over the years through its enduring capacity to concurrently handle all these evaluations and their updates. A single problematic isotope can obstruct the system’s operation, underscoring the necessity of these qualities in the processing of certified nuclear data for nuclear reactor physics. For over 51 years, the Njoy nuclear data processing system (Fig. 1) has played a pivotal role in supporting certified and qualified Boltzmann and MC codes’ missions, including neutral particle transport, regulatory licensing processes, advanced fission and fusion reactor design, safety assessment, criticality safety benchmarking, stockpile stewardship modeling, radiation shielding, and nuclear waste management. Throughout these years, Njoy has remained limited to neutron and photon transport. Our introduction of ELECTR (Fig. 1) represents the first expansion of the system’s functionalities to accommodate light charged particles^130,131^. ELECTR operates under two distinct modes: ENDF^120^ and CEPXS^132^. This paper concerns the extension of CEPXS-mode capabilities for VHEE and UHEE beams. The multigroup CEPXS formalism from the U.S. Sandia National Laboratory has never been validated beyond $\text{20 MeV}$^104^. The objective of this paper is to demonstrate that an extension beyond its design limit of $\text{100 MeV}$, announced at its release in October 1989^132^, is possible. It is important to place this effort into context. The CEPXS-mode in ELECTR serves as a foundation for the development of the ENDF-mode. The Lorence–Morel–Valdez postulates^132^, that we will discuss in what follows, provide a basis for a first application of multigroup theory to ENDF data. For this reason, we propose this final step: to extend the state-of-the-art to VHEE and UHEE beams prior to any release of a fully operational open-source ENDF-mode. Unlike the last work proposed for the CONV-RT^104^, we are now proposing a detailed presentation of the analytical cross sections implemented in the CEPXS mode in VHEE- and UHEE-RT.

### VHEE multigroup theory

Let *g*, *n* and $\varvec{\vec {r}}$ be the VHEE/UHEE energy group, its discrete ordinate direction and position, respectively. If $\psi ^e$ (e$^-$/cm$^2$/s) designates the electron flux, $\Sigma _t$ (cm$^{-1}$) the macroscopic total cross section, $\Sigma _{l}^{x}$ (cm$^{-1}$) the *l*th Legendre macroscopic scattering cross section coefficient associated to interaction *x* and $Q^{e,\text {ext.}}$ (e$^-$/cm$^3$/s) the external source, the $S_N$ multigroup form of the first-order BFP equation is given by:

1 $$\begin{aligned}{} & {} \left[ \hat{\Omega }_{n}\cdot \mathbf {\nabla }+\Sigma _{t,g}(\varvec{\vec {r}})\right] \psi _{g,n}^e(\varvec{\vec {r}}) = \sum \limits _{x}^{} \sum \limits _{l=0}^{L} \sum \limits _{m=-l}^{+l} \sum \limits _{g'}^{G} \ \Sigma _{l,g'\rightarrow g}^{x,0}(\varvec{\vec {r}}) R_{l}^{m}(\hat{\Omega }_n) \psi _{l,g'}^{m}(\varvec{\vec {r}})\nonumber \\{} & {} + \frac{\partial }{\partial E_{g}} \beta _{g}(\varvec{\vec {r}}) \psi _{g,n}^e(\varvec{\vec {r}}) + \frac{1}{2} \alpha _{g}(\varvec{\vec {r}}) \left[ \frac{\partial }{\partial \mu _{n}}\left( 1-\mu _{n}^2\right) \frac{\partial }{\partial \mu _{n}} + \frac{1}{1-\mu _{n}^2} \frac{\partial ^2}{\partial \phi _{n}^2} \right] \psi _{g,n}^e(\varvec{\vec {r}}) + Q_{g,n}^{e,\text {ext.}}(\varvec{\vec {r}}). \end{aligned}$$

where $R_l^m$ denote the real spherical harmonics. The electron direction ($\hat{\Omega }_{n}$) is uniquely defined by the polar angle cosine ($\mu _n\in [-1,+1]$) and the azimuthal angle ($\phi _n\in [0,2\pi ]$). Total and scattering cross sections in Eq. (1) are restricted to catastrophic collisions while stopping powers ($\beta _g$ [MeV/cm]) and momentum transfers ($\alpha _g$ [cm$^{-1}$]) are restricted to soft ones. The *m*th moment of the *l*th Legendre order group-to-group microscopic transfer cross section ($\text {barns}\cdot \text {MeV}^m$) is given by:

2 $$\begin{aligned} \sigma _{l,g'g}^{x,m}(\varvec{\vec {r}}) = \int _{-1}^1 \text {d}\mu P_{l}(\hat{\Omega }'\cdot \hat{\Omega }) \int _{E_g'}^{E_{g'+1}} dE' \int _{I_{g}^{\textrm{x}}} dE \ \sigma _{\textrm{x}}(E' \rightarrow E,\hat{\Omega }'\cdot \hat{\Omega }) (E')^m. \end{aligned}$$

Two quantities are needed for each *x*, the DDSC ($\sigma _x$ in Eq. 2) and the VHEE/UHEE transfer groups ($I_{g}^{\textrm{x}}$ in Eq. 2).

The ELECTR [CEPXS-mode] considers the ionization ($x=i$) as a two-body mechanism, made on a free electron. No binding energy is involved in the process. Two particles emerge from a typical CEPXS *i*-event; the scattered primary electron and the delta ($\delta$) ray. The *i*-DDSC is that of Møller. Unlike Class-I and-II MC codes, no straggling model is needed for the Njoy-Dragon chain. The energy distributions are derived directly from Møller’s DDSC. The scattering angles for the primary and $\delta$ electrons are determined from the *i*-collision kinematics, with $\mu _{e/\delta }^i=\sqrt{e_{e/\delta }(e'_e+2)/e'_e(e_{e/\delta }+2)}$. $e_{e/\delta }$ energies are in reduced units. One can verify that both $\mu _e^i$ and $\mu _\delta ^i$ are forward peaked. For this reason, some MC codes assume that $\mu _e^i=1$ and account for the primary angular deflection by a modification to the nuclear elastic kernel. This is not the case for the Njoy-Dragon chain. The same kinematics provide $e_\delta \in [0,e'_e/2]$. However, because of (1) the singularity of Møller’s DDSC (Eqs. 3–5) at $e_\delta =0$; and (2) the quantum wave-particle duality at low energy, we restrict $e_\delta \in [e_c,e'_e/2]$. This immediately implies $e_e\in [e'_e/2,e'_e-e_c]$. The cutoff energy ($e_c$) is thus identified as the lowest energy a $\delta$-ray could have as a result of a catastrophic *i*-collision. In multigroup theory, the ELECTR [CEPXS-mode] follows Lorence’s^132^ and Morel’s^133^ proposal to consider a catastrophic collision, an *i*-event in which the scattered VHEE does not appear in two adjacent groups, i.e., $e_c=e'_e-e_{e}^{g'-2}$, $\forall e'_e \in [e_e^{g'},e_e^{g'+1}]$. Consequently, $e_e\in [e'_e/2,e_{e}^{g'-2}]$. Integral boundaries for multigroup transfer matrices can be deduced easily by crossing the Njoy[ELECTR] user group structure with the kinematics restrictions.

3 $$\begin{aligned}{} & {} \sigma _{l,g'g}^{i,m}(\varvec{\vec {r}}) = \sigma _{l,g'g}^{ee,m}(\varvec{\vec {r}}) + \sigma _{l,g'g}^{e\delta ,m}(\varvec{\vec {r}}) \end{aligned}$$

4 $$\begin{aligned}{} & {} \sigma _{l,g'g}^{ee,0}(\varvec{\vec {r}}) = \frac{1}{\Delta e_e^{g'}} \int \limits _{e_e^{g'}}^{e_e^{g'+1}} \!\! \!\! \textrm{d}e'_e \!\! \!\! \!\! \!\! \int \limits _{\max (e_e^g,\frac{e_e'}{2})}^{e_{e}^{g+1}} \!\!\!\! \!\!\!\! \!\! \!\! \;\;\textrm{d}e_e \ H\left[ e_{e}^{g+1}-\max (e_e^{g},\frac{e_e'}{2})\right] P_l(\mu _e) \left( \frac{r_0}{\beta '_e}\right) ^2\nonumber {} & {} \left[ \frac{1}{e_\delta ^2}+\frac{1}{e_e^2}+\frac{1}{(e_e'+1)^2}-\frac{(2e'_e+1)}{(e'_e+1)^2 e_e e_\delta } \right] , \nonumber \\{} & {} \forall e_e^{g+1} \in \{e_e^{g'-2},...,e_e^{g'=1}\}. \end{aligned}$$

5 $$\begin{aligned}{} & {} \sigma _{l,g'g}^{e\delta ,0}(\varvec{\vec {r}}) = \frac{1}{\Delta e_e^{g'}} \int \limits _{e_e^{g'}}^{e_e^{g'+1}} \!\! \!\! \textrm{d}e'_e \!\! \!\! \int \limits _{e_\delta ^g}^{\min (e_\delta ^{g+1},\frac{e_e'}{2})} \!\!\!\! \!\!\!\! \!\! \!\! \textrm{d}e_\delta \ H\left[ \min \left( e_\delta ^{g+1}-\frac{e_e'}{2}\right) -e_\delta ^g\right] P_l(\mu _\delta ) \left( \frac{r_0}{\beta '_e}\right) ^2\nonumber {} & {} \left[ \frac{1}{e_\delta ^2}+\frac{1}{e_e^2}+\frac{1}{(e_e'+1)^2}-\frac{(2e'_e+1)}{(e'_e+1)^2 e_e e_\delta } \right] , \nonumber \\{} & {} \forall e_\delta ^{g+1} \in \{e_e^{g'-2},...,e_e^{g'=1}\}. \end{aligned}$$

where $\beta _e'$ refers to the incident electron’s velocity in the speed of light units and $r_0$ to the classical electron radius. *H* is the Heaviside function. From Eqs. (4)–(5), it is clear that the degree of correlation between the $\delta$ and primary emission spectra is dictated by the user multigroup structure in Njoy. Moreover, the minimum $\delta$-ray energy cannot fall below the Njoy user-selected lower energy limit. Several authors, including Morel^133^, Lorence^132^, Olbrant^134^, Cullen^135–137^ and Salvat^138^, recommend a $\text{1 keV}$ transport cutoff. This is also the default value used by Njoy-Dragon for CONV-RT^104^, VHEE-RT and UHEE-RT. A $\text{100 eV}$ limit is possible only in the ELECTR [ENDF-mode]. Otherwise, in the CEPXS-mode, if $e_\delta <\text{1 keV}$, only the primary electron undergoes deflection, and the $\delta$-ray is not produced. In this case, the associated soft energy loss is addressed by the Fokker-Planck continuous slowing down (CSD) operator (Eq. 1).

The catastrophic production of VHEE/UHEE bremsstrahlung electrons ($x=b$) is possible in an electric atomic field or a nuclear one. Electrons are emitted in the incident particle’s direction ($\mu _e^b=1$), while photons follow a Sommerfield angular distribution^139^. The *b*-DDSC is that of Berger–Seltzer^140^ based on Koch–Motz developments^141,142^ and Born’s assemblies. Like Møller, Berger–Seltzer’s DDSC shows a singularity at $e_\gamma =0$, so the same cutoff $e_c$ must be applied for the catastrophic radiative emission. Moreover, a high-frequency limit ($e_\gamma ^m$) must be defined to avoid the divergence of the atomic-dependent Elwert screening factor $f_e$ when $e_\gamma =e'_e$. We have:

6 $$\begin{aligned} \begin{aligned}{}&\sigma _{l,g'g}^{b,0}(\varvec{\vec {r}}) = \frac{1}{\Delta e_e^{g'}} \int \limits _{e_e^{g'}}^{e_e^{g'+1}} \!\! \!\! \textrm{d}e'_e \!\! \!\! \!\! \!\! \int \limits _{\max (e_{e}^{g},e_{e}'-e_\gamma ^m)}^{e_{e}^{g+1}} \!\!\!\! \!\!\!\! \!\! \!\! \textrm{d}e_e \ \xi _r(e'_e) f_e(e'_e \rightarrow e_\gamma ) \left\{ \sigma _1(e'_e \rightarrow e_\gamma ) + \sigma _2(e'_e \rightarrow e_\gamma )\right. \\&\left. - \sigma _3(e'_e \rightarrow e_\gamma ) + \omega (e'_e) \left[ \sigma _4(e'_e \rightarrow e_\gamma ) \right. \right. \left. \left. - \sigma _2(e'_e \rightarrow e_\gamma )\right] \right\} , \ \forall e_e^{g+1} \in \{e_e^{g'-2},...,e_e^{g'=1}\}. \end{aligned} \end{aligned}$$

$\sigma _1$ and $\sigma _2$ refer to Sauter–Gluckstern–Hull^143,144^ unscreened and screened scattering kernels, respectively. $\sigma _3$ refers to Schiff^145^ unrelativistic kernel, while $\sigma _4$ to Olsen–Maximon’s^146^ Coulomb correction for small angles. The first three kernels are derived in the Born approximation, while $\sigma _4$ goes beyond that to include a Sommerfeld–Mauve wave function. The DSCs assembly (Eq. 6) is made in such a way to avoid discontinuities arising from abrupt switching from one kernel to another. $\xi _r$ represents the Berger–Seltzer atomic-dependent correction factor. $\omega (e'_e)$ is a weighting function introduced by Berger and Seltzer^140^ to switch off Coulomb correction at low energies where it becomes unreliable.

7 $$\begin{aligned}{} & {} \sigma _1(e'_e\rightarrow e_\gamma )= \frac{Z(Z+1)r_0^2}{137e_\gamma } \frac{p_e}{p_e'} \left\{ \frac{4}{3} - 2 e_t e_s \left( \frac{p_e^2+{p_e'}^2}{p_e^2 {p_e'}^2} \right) + \xi _e' \frac{e_t}{{p_e'}^3} + \xi _e \frac{e_t}{p_e^3} + \frac{\xi _e \xi _e'}{p_e p_e'} \right. \nonumber \\{} & {} \left. + \mathscr {L} \left[ \frac{8}{3} \frac{e_t e_s}{p_e p_e'} + \frac{e_\gamma ^2}{p_e^3 {p_e'}^3} \left( e_t^2 e_s^2 + {p_e'}^2 p_e^2 \right) + \frac{e_\gamma }{2p_e' p_e} \left[ \left( \frac{e_t e_s + {p_e'}^2 }{{p_e'}^3} \right) \xi _e' - \left( \frac{e_t e_s + p_e^2 }{p_e^3} \right) \xi _e + \frac{2 e_\gamma e_t e_s}{p_e^2 {p_e'}^2} \right] \right] \right\} \end{aligned}$$

8 $$\begin{aligned}{} & {} \sigma _2(e'_e\rightarrow e_\gamma )= \frac{4 Z(Z+1)r_0^2}{137e_\gamma } \left[ \left( 1+ \frac{e_s^2}{e_t^2} \right) \left( \frac{1}{4} \Phi _1(\zeta ) - \frac{1}{3} \log (Z) \right) - \frac{2}{3} \frac{e_s}{e_t} \left( \frac{1}{4} \Phi _2(\zeta ) - \frac{1}{3} \log (Z) \right) \right] \end{aligned}$$

9 $$\begin{aligned}{} & {} \sigma _3(e'_e\rightarrow e_\gamma )= \frac{4 Z(Z+1)r_0^2}{137e_\gamma } \left( 1-\frac{2}{3} \frac{e_t}{e_s} + \frac{e_t^2}{e_s^2} \right) \left[ \log \left( \frac{2 e_t e_s}{e_\gamma } \right) -\frac{1}{2} \right] \end{aligned}$$

10 $$\begin{aligned}{} & {} \sigma _4(e'_e\rightarrow e_\gamma )= \frac{4 Z(Z+1)r_0^2}{137e_\gamma } \left[ \left( 1+ \frac{e_s^2}{e_t^2} \right) \left( \frac{1}{4} \Phi _1(\zeta ) - \frac{1}{3} \log (Z) - f(Z) \right) \right. \nonumber \\{} & {} \left. - \frac{2}{3} \frac{e_s}{e_t} \left( \frac{1}{4} \Phi _2(\zeta ) - \frac{1}{3} \log (Z) - f(Z) \right) \right] \hspace{2em} \end{aligned}$$

$e_t$ refers to the total incident electron energy. $e_s$ denotes the total energy lost during the b-event. *p* is the particle’s momentum in reduced units, $\mathscr {L}=2 \log [(e_t e_s + p_e p'_e - 1)/e_\gamma ]$ and $\xi _e'=\log [(e_s+p_e')/(e_s-p_e')]$. $\Phi _1$ and $\Phi _2$ are the screening factors. $\zeta$ is a b-spectrum dependent factor. *f*(*Z*) is a Z-dependent empirical correction factor. The nuclear contribution to bremsstrahlung is taken into account in the CEPXS-mode by modifying $Z(Z+1)$ by $Z^2$. One should note that all scattering kernels (Eqs. 7–10) vanish in the high-frequency limit. For the latter, the CEPXS-mode uses Berger–Seltzer extrapolations for consistency between the theoretical work of Fano–Koch–Motz^147^, McVoy–Fano^148^, Jarbur–Pratt^149^ and the experimental data.

Elastic scattering ($x=e$) is predominantly treated in a nuclear field. The electron changes direction without any loss of energy. In the non-relativistic domain ($E'_e\le \text{256 keV}$), the CEPXS-mode implements Riley’s DDSC ([*R*]) while, for the relativistic domain, a Molière-screened Mott DDSC ([*M*]) is used . For the latter domain, within the Born approximation to the Lippmann–Schwinger scattering state, we have^150^:

11 $$\begin{aligned} \sigma _{l,g'g}^{e[M],0}(\varvec{\vec {r}}) = 2 \pi r_0^2 Z^2 \delta _{g'g}\int _{-1}^{+1} \text {d}\mu _e \ P_l(\mu _e) \left[ \frac{e_e+1}{e_e(e_e+2)}\right] ^2 \left[ \frac{\chi (\mu _e,e_e)}{(1-\mu _e+2\eta _e)^2} + \frac{2}{\sqrt{2}} \pi Z \frac{\beta _e}{137} \frac{\nu _e}{\root 3 \of {1-\mu _e+2\eta _e}} \right] . \end{aligned}$$

$\eta _e$ is given by Nigam’s^151,152^ improved Molière theory and computes the orbital electrons induced-nuclear charge screening. $\chi (\mu _e,e_e)$ refers to the Mott–Rutherford ratio cross section as evaluated by McKinley and Feshback^153^ for low *Z*, extended by Doggett and Spencer^154^ for medium and high *Z* and tabulated by Birkhoff and Sherman^155^ for discrete scattering angles and incidence energies. $\nu _e$ is an empirical energy-dependent correction factor. Unlike the ENDF-mode, the CEPXS-mode in ELECTR avoids using angular quadrature for Eq. 11 integration. A Goudsmit–Saunderson (GS)^156,157^ moment-based semi-analytical approach is used for Eq. (11). The Mott-based GS moments are given by:

12 $$\begin{aligned}{} & {} \mathscr {G}_{l}^{g}[M] = 4 \pi ^2 r_0^2 Z^2 \delta _{g'g}\int _{-1}^{+1} \text {d}\mu _e \ \left[ 1-P_l(\mu _e)\right] \left[ \frac{e_e+1}{e_e(e_e+2)}\right] ^2 \left[ \frac{\chi (\mu _e,e_e)}{(1-\mu _e+2\eta _e)^2} + \frac{2}{\sqrt{2}} \pi Z \frac{\beta _e}{137} \frac{\nu _e}{\root 3 \of {1-\mu _e+2\eta _e}} \right] . \end{aligned}$$

13 $$\begin{aligned}{} & {} \mathscr {G}_l^g[M] = \sigma _{0,g'g}^{e,0}(\varvec{\vec {r}}) - \sigma _{l,g'g}^{e,0}(\varvec{\vec {r}}), \ \forall l \in \{1,...,L\}. \end{aligned}$$

A high-order expansion of $\mathscr {G}_l$ is required for forward-peaked multiple scattering (e.g., $L=60$ for $\mu _e=0.986286 \ (9.5^\circ )$^158^). To avoid the slowly converging Legendre series in the deflection angle, Spencer’s numerical substitution is introduced^159^: $\Xi (\mu _e)=\sum _{j=1}^J \Xi _j \root j \of {1-\mu _e+2\eta _e}$. This representation has been demonstrated to be accurate to within $1\%$ with $J=5$. The latter is referred to as the five Bartlett–Watson tabulated cosine angles^154^ and is utilized in the CEPXS-mode. Combining Eqs. 11–13 and $\Xi (\mu _e)$, $\mathscr {G}_l^g[M]$ moments can be redefined as a linear combination of Spencer’s integrals for Mott scattering^160^:

14 $$\begin{aligned}{} & {} \mathscr {G}_l^g[M] = 4 \pi ^2 r_0^2 Z^2 \left[ \frac{e_e+1}{e_e(e_e+2)}\right] ^2 \delta _{g'g} \left[ \mathscr {P}_l^{-2} + \frac{\pi Z \beta _e}{137\sqrt{2}} \chi _e \mathscr {P}_l^{-3/2} \sum _{j} \Xi _j \mathscr {P}_l^{j-3}[M] \right] . \end{aligned}$$

15 $$\begin{aligned}{} & {} \mathscr {P}_l^{m}[M] = \int _{-1}^{+1} \text {d}\mu _e \left( 1-\mu _e+2\eta _e\right) ^m \left[ 1-P_l(\mu _e)\right] , \ \forall l. \end{aligned}$$

where recursion forms can be derived from the Legendre polynomials as proposed by Spencer^159^:

16 $$\begin{aligned} \mathscr {P}_l^{m+1}[M] = \mathscr {P}_1^{m}[M] - \frac{l}{2l+1} \mathscr {P}_{l-1}^{m}[M] + (1+2\eta _e) \mathscr {P}_l^{m}[M] - \frac{l+1}{2l+1} \mathscr {P}_{l+1}^{m}[M], \ \forall l. \end{aligned}$$

It is worth noting that the accuracy of Eqs. (11)–(16) remains less than that achieved by Brown’s (1961) method^158^, which involves a direct numerical resolution of the Dirac equation with a screened Coulomb potential. Moreover, the Elsepa kernel^161,162^, designed for relativistic Dirac partial-wave elastic scattering and implemented within the Penelope framework^138^, is expected to yield further improved accuracy. Conversely, the Riley DDSC can be derived from a partial-wave expanded Dirac equation resolution in a Poisson spherical central atomic static potential field^163^. This exact solution can be fitted using 12*g*-dependent parameters^164^.

17 $$\begin{aligned} \sigma _{l,g'g}^{e[R],0}(\varvec{\vec {r}}) = D \delta _{g'g} \sum _{m=1}^{4} A_m(e_e') \left[ 1-\mu _e + 2 B(e_e') \right] ^{-m} + \sum _{n=0}^6 C_n(e_e') P_n(\mu _e). \end{aligned}$$

where the first sum is developed to fit the small-angle deflections and the second one to fit moderate and large-angle tail. *B* is a screening parameter and $A_m$ and $C_n$ are in ${\text{\AA }}/\text {Sr}$. The idea of the fit is in response to the need in both MC and BFP for an accurate, rapid, and analytic scattering kernel at low energies. Equation (17) is validated against Fink and Kessler^165^ absolute small-angle measurements and Ibres-Vainshtein^166^ Born approximation results. The Riley-based GS moments and Spencer associated functions are given by^159^:

18 $$\begin{aligned} \mathscr {G}_l^{g,R}(\varvec{\vec {r}})= & {} 2 \pi D \left[ \sum _{m=1}^{4} A_m(e_e') \mathscr {P}_l^{-m}[R] + C_0(e'_e) - C_l(e'_e) \right] . \end{aligned}$$

19 $$\begin{aligned} \mathscr {P}_l^{m}[R]= & {} \int _{-1}^{+1} \text {d}\mu _e \left( 1-\mu _e+2B\right) ^m \left[ 1-P_l(\mu _e)\right] , \ \forall l. \end{aligned}$$

Finally, both Riley and Mott’s DDSCs follow a transport correction to bring back the highly-forward peaked scattering kernels reducible to a lower-order Legendre expansion^167^.

20 $$\begin{aligned} \bar{\sigma }_{l,g'g}^{e[M/R],0}(\varvec{\vec {r}}) = \sigma _{l,g'g}^{e[M/R],0}(\varvec{\vec {r}}) - \sigma _{L,g'g}^{e[M/R],0}(\varvec{\vec {r}}), \ \forall l. \end{aligned}$$

**Figure 2:**
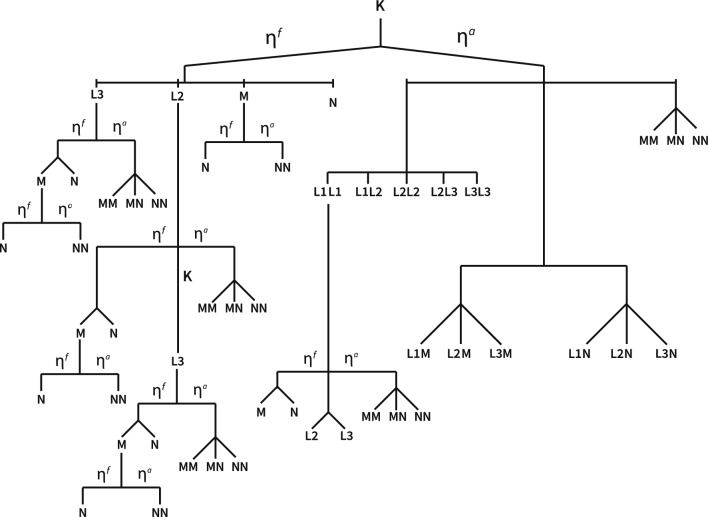
Simplified partial relaxation cascades from *K*, $L_1$, $L_2$, $L_3$, *M* and *N* shells and subshells in ELECTR [CEPXS-mode]. $\eta _a$ and $\eta _f$ refer, respectively, to Auger and fluorescence relaxation efficiency. Relaxation stops when the vacancy is transferred to the outermost subshell. The line radiation for a given relaxation is obtained by considering a conditioned multiplication of the probabilities of all the involved branches. The complete cascade is depicted by replicating the de-excitations at each call of the subshell. For illustration purposes, the duplication in this figure is demonstrated only for a few branches.

Unlike the ENDF-mode, the Auger ($x=a$) and fluorescence ($x=f$) relaxation cascades in the CEPXS-mode are uncorrelated with *i*-events. Consequently, an impact ionization cross-section is separately introduced in the CEPXS-mode. All *a*- and *f*-events involving secondary emission below the Njoy-Dragon chain cutoff ($e_c$) are excluded. As a result, only *K*, $L_1$, $L_2$, $L_3$, *M*, and *N* shells and subshells, involving $Z>10$, $Z>27$, $Z>29$, $Z>29$, $Z>51$, and $Z>84$ elements, are considered as cascade candidates. The energy of the emitted particle is determined by the difference in binding energies of the involved subshells. The Auger electron emission is isotropic ($l=0$), and thus, the Auger transfer matrix is given by:

21 $$\begin{aligned} \sigma _{0,g'g}^{a,0} = \sum _{j=1}^{N_{s}} \sum _{k=1}^{N_{t}} \delta _{g'g_k} \upsilon ^j_e \eta _{jk}^{a} \sigma _{0,g'g}^{j,0} \end{aligned}$$

where *j* refers to the ionized shell and *k* to the allowed line radiation. The maximum number of subshells ($N_s$) and transition lines ($N_t$) are restricted to 5 and 28, respectively. $\upsilon ^j_e$ denotes the number of electrons in the *j*th subshell. $\eta _{jk}^a$ indicates the relaxation efficiency for a *k*th Auger emission following the *j*th subshell ionization event. The $g_k$ transfer group arises from loop convergence, assigning the emitted Auger electron to one of the $\textsc {Njoy}$ user-selected groups. Given that $e_b^j$ represents the *j*th subshell binding energy in reduced units, the Gryzinski^168^ impact ionization cross section is expressed as:

22 $$\begin{aligned} \sigma _{0,g'g}^{j,0}(\varvec{\vec {r}}) = \frac{\pi }{\Delta e_e^{g'}} \int _{e_e^{g'}}^{e_e^{g'+1}} \textrm{d}e'_e \left( \frac{r_0}{e_b^j}\right) ^2 \frac{\beta _j^2}{\beta _e^2} \root 3 \of {\frac{\beta _e^2}{\beta _e^2+\beta _j^2 - \beta _e^2\beta _j^2}} \left[ 1+\frac{2}{3} \left( 1-\frac{e_b^j}{2e'_e}\right) \log \left( 2.7+\sqrt{\frac{e_e'}{e_b^j}-1}\right) \right] \root 3 \of {1-\frac{e_b^j}{e_e'}}, \end{aligned}$$

where $\beta _j=\sqrt{e_b^j (e_b^j+2)}/(e_b^j+1)$. Fig. 2 shows the complete cascade allowed by ELECTR [CEPXS-mode]. All *N* shells intervene with $e_b^N=0$. The relaxation process, triggered by an inner vacancy, ceases if the latter is transferred to the outermost shell. Each relaxation cascade efficiency (radiative or non-radiative) is computed from a multiplication of the specific conditional branch probabilities.

For a given incidence group, the total catastrophic cross-section (Eq. 1) is obtained by first integrating all scattering kernels (Eqs. 4, 6–10, 12–16, and 17–19) over all Legendre orders and all possible emission energies, and then summing the resultant values. This second integral must adhere to the definition of the catastrophic jump, the Møller non-divergence condition, the associated kinematic restrictions, and the bremsstrahlung high-frequency limit.

23 $$\begin{aligned} \sigma _{t,g'}(\varvec{\vec {r}})= \sum _g \ \sigma _{0,g'g}^{ee,0} + \sigma _{0,g'g}^{b,0} + \sigma _{0,g'g}^{e[M],0}+\sigma _{0,g'g}^{e[R],0}. \end{aligned}$$

In ELECTR [CEPXS-mode], soft VHEE/UHEE stopping powers ($\beta _g$) for both radiative and non-radiative events are derived neither from single-event cross sections nor from down-scattering in adjacent energy groups. Instead, Berger’s collisional ($\beta ^c_{t}$) and radiative ($\beta ^r_{t}$) total stopping power tabulations are extrapolated at the incident VHEE/UHEE midpoint group ($e_{g'}^m$) and corrected for the plasmon effect using a Lorence correction. Soft stopping powers are obtained by subtracting catastrophic stopping powers, derived by integrating the first moment of group-to-group transfer cross sections across all Legendre orders and energy losses, from $\beta ^c_{t}$ and $\beta ^r_{t}$. Soft collisions do not involve energy loss straggling or angular deflection.

24 $$\begin{aligned} \beta _{g'}^{c}= & {} \beta ^c_t(e_{g'}^m) - \frac{m_ec^2}{\Delta e_e^{g'}} \int _{e_e^{g'}}^{e_e^{g'+1}} \textrm{d}e'_e \int _{e_e'/2}^{e_{e}^{g+1}} \textrm{d}e_e \ H\left[ e_{e}^{g+1}-\max (e_e^{g},\frac{e_e'}{2})\right] (e'_e-e_e) \sigma _M(e'_e \rightarrow e_\delta ), \ \nonumber \\{} & {} \forall e_e^{g+1} \in \{e_e^{g'-1},...,e_e^{g'=1}\}. \end{aligned}$$

25 $$\begin{aligned} \beta _{g'}^{r}= & {} \beta ^r_t(e_{g'}^m) - \frac{m_e c^2}{\Delta e_e^{g'}} \int _{e_e^{g'}}^{e_e^{g'+1}} \textrm{d}e'_e \int _{e_{e}'-e_\gamma ^m}^{e_{e}^{g+1}} \textrm{d}e_e \ (e'_e-e_e) \sigma _{b} (e'_e \rightarrow e_\gamma ), \forall e_e^{g+1} \in \{e_e^{g'-1},...,e_e^{g'=1}\}. \end{aligned}$$

Soft collisions as well as catastrophic *i*-, *b*- and *a*-events define the energy deposition cross section. We can infer catastrophic energy deposits from local absorptions. Therefore, the total energy deposition cross section can be expressed as follows:

26 $$\begin{aligned} \mathscr {E}^t_{g'} = \sum _{x\in \{ee,e\delta ,b\}} \left[ \sigma _{t,g'}^{x,1} - \sum _{h=1}^{g'-2} \sigma _{0,hg}^{x,1} \right] + \sigma _{0,g'g}^{a,1} + \beta _{g'}^c + \beta _{g'}^r. \end{aligned}$$

Once the MATXS library is produced, the numerical solution of Eq. 1 can be found using the Dragon-5 solver. First, we assume a continuous variation of the flux over each group and spatial domain and a linear variation of the angular flux and scattering source over each group. Next, by (1) applying the Galerkin method of weighted residuals; (2) substituting the flux and scattering source with a second-order energy expansion; (3) multiplying Eq. (1) (in 1D) by normalized Legendre polynomials; (4) integrating over the appropriate support $E\in [-1/2,+1/2]$; and (5) canceling the first moments of the flux and scattering sources, it can be shown that:

27 $$\begin{aligned}{} & {} \left[ \mu _n \frac{\partial }{\partial x}+\Sigma _{t,g}(x)\right] \psi _{g,n}^e(x) = \sum \limits _{x}^{} \sum \limits _{l=0}^{L} \sum \limits _{g'}^{G} \frac{2l+1}{2} P_l(\mu _n) \Sigma _{l,g'\rightarrow g}^{x,0}(x) \psi _{l,g'}^{e}(x)\nonumber \\{} & {} -\frac{1}{\Delta E_{g}} \left[ \beta _{g}^+(x) \psi _{g,n}^{e,+}(x) - \beta _{g}^-(x) \psi _{g,n}^{e,-}(x) \right] + \frac{1}{2} \alpha _{g}(x) \frac{\partial }{\partial \mu _{n}}\left( 1-\mu _{n}^2\right) \frac{\partial }{\partial \mu _{n}} \psi _{g,n}^e(x) + Q_{g,n}^{e,\text {ext.}}(x). \end{aligned}$$

with the energy propagation relation given by:

28 $$\begin{aligned} \beta _{g}^+(x) \psi _{g,n}^{e,+}(x) + \beta _{g}^-(x) \psi _{g,n}^{e,-}(x) = \left[ \beta _{g}^+(x) + \beta _{g}^-(x) \right] \psi _{g,n}^e(x). \end{aligned}$$

where $\beta _{g}^\mp (\varvec{\vec {r}})$ and $\psi _{g,n}^{e,\mp }(\varvec{\vec {r}})$ refer to the soft stopping powers and angular fluxes at the *g*th group upper and lower boundaries, respectively. The base cosine angles ($\mu _n$) and associated weights ($w_n$) are given by an *N*-point Gauss–Legendre quadrature. Prior to inverting the system, spatial discretization is performed. Denoting $\psi _{g,n}^{e,i^\pm }$ and $\psi _{g,n}^{e,i}$ as the electron flux’s mesh-edge and mesh-centered values for a specific sub-mesh *i*, the same procedure applied in energy can be employed. By (1) expanding the flux and sources’ spatial expansions of order *M* using normalized Legendre polynomials; (2) weighting Eqs. (27) and (28) with normalized (in space) Legendre moments; (3) integrating Eqs. (27) and (28) over each submesh region to obtain the spatial moments of Eq. (27) and spatial Legendre moments of the slowing down angular flux (Eq. 28); and (4) applying a diamond-difference discretization of the electron flux:

29 $$\begin{aligned} \psi _{g,n}^{e,i^\mp }= {\left\{ \begin{array}{ll} 2 \psi _{g,n,i}^{[0]} - \psi _{g,n}^{e,i^\pm } \, &{}\forall M=0 \\ \psi _{g,n}^{e,i^\pm } \mp 2\sqrt{3} \psi _{g,n,i}^{[1]} \, &{}\forall M=1 \\ 2 \psi _{g,n,i}^{[0]} +2\sqrt{5} \psi _{g,n,i}^{[2]} - \psi _{g,n}^{e,i^\pm } \, &{}\forall M=2 \\ ... \\ \end{array}\right. }\, \end{aligned}$$

With the variable change $x_i=1/\Delta x_i [x-1/2(x_{i+1/2}+x_{i-1/2})]$, Bienvenue and Hébert^169^ showed that the discretized 1D BFP equation can be resolved recursively by initiating with known entering fluxes (generally a vacuum boundary condition) and computing the other mesh-edge and flux moments:

30 $$\begin{aligned} \psi _{g,n,i}^{[0]}= & {} \frac{\Delta x_i \widetilde{L}_{g,n,i}^{[0]} + 2 |\mu _n| \psi _{g,n}^{e,i^\pm }}{\Delta x_i \widetilde{\Sigma }_{t,g}^i + 2 |\mu _n|}, \ \forall M=0. \end{aligned}$$

31 $$\begin{aligned}{} & {} \begin{bmatrix} \psi _{g,n,i}^{[0]} \\ \psi _{g,n,i}^{[1]} \end{bmatrix} = \begin{bmatrix} \Delta x_i \widetilde{\Sigma }_{t,g}^i &{} 2\sqrt{3}\mu _n \\ 2\sqrt{3}\mu _n &{} -\Delta x_i \widetilde{\Sigma }_{t,g}^i-6 |\mu _n| \end{bmatrix}^{-1} \ \begin{bmatrix} \Delta x_i \widetilde{L}_{g,n,i}^{[0]} \\ -\Delta x_i \widetilde{L}_{g,n,i}^{[1]} + 2\sqrt{3}\mu _n \psi _{g,n}^{e,i^\pm } \end{bmatrix}, \ \forall M=1. \end{aligned}$$

32 $$\begin{aligned}{} & {} \begin{bmatrix} \psi _{g,n,i}^{[0]} \\ \psi _{g,n,i}^{[1]} \\ \psi _{g,n,i}^{[2]} \end{bmatrix} = \begin{bmatrix} \Delta x_i \widetilde{\Sigma }_{t,g}^i+ 2 |\mu _n| &{} 0 &{} 2 \sqrt{5} |\mu _n| \\ 0 &{} -\Delta x_i \widetilde{\Sigma }_{t,g}^i &{} -2 \sqrt{15} \mu _n \\ 2 \sqrt{5} |\mu _n| &{} -2 \sqrt{15} \mu _n &{} \Delta x_i \widetilde{\Sigma }_{t,g}^i+ 10 |\mu _n| \end{bmatrix}^{-1} \begin{bmatrix} \Delta x_i \widetilde{L}_{g,n,i}^{[0]} + 2 |\mu _n| \psi _{g,n}^{e,i^\pm } \\ -\Delta x_i \widetilde{L}_{g,n,i}^{[1]} \\ \Delta x_i \widetilde{L}_{g,n,i}^{[2]} + 2\sqrt{5} |\mu _n| \psi _{g,n}^{e,i^\pm } \end{bmatrix}, \ \forall M=2. \end{aligned}$$

where,

33 $$\begin{aligned} \widetilde{\Sigma }_{t,g}^i= & {} \Sigma _{t,g}^i + \frac{1}{\Delta E_g} \left( \beta _{g,i}^- + \beta _{g,i}^+ \right) . \end{aligned}$$

34 $$\begin{aligned} \widetilde{L}_{g,n,i}^{[\alpha ]}= & {} \sum \limits _{x}^{} \sum \limits _{l=0}^{L} \sum \limits _{g'}^{G} \frac{2l+1}{2} P_l(\mu _n) \int _{-1/2}^{+1/2} \textrm{d}x_i \widetilde{P}_\alpha (x_i) \Sigma _{l,g'g,i}^{x,0} \psi _{l,g'}^{e,i} + \frac{2}{\Delta E_g} \beta _{g,i}^- \psi _{g,n,i}^{-[\alpha ]} \end{aligned}$$

35 $$\begin{aligned} \psi _{g,n,i}^{[\alpha ]}= & {} \int _{-1/2}^{+1/2} \textrm{d}x_i \widetilde{P}_\alpha (x_i) \psi _{g,n}^{e,i}, \ \psi _{g,n,i}^{\mp [\alpha ]} = \int _{-1/2}^{+1/2} \textrm{d}x_i \widetilde{P}_\alpha (x_i) \psi _{g,n}^{e,i\mp }. \end{aligned}$$

Upon concluding each inner loop, the electron flux angular and spatial Legendre moments are deduced from Eq. (36). Utilizing these values, the subsequent iteration’s spatial and angular Legendre moments, $\widetilde{L}_{g,n,i}$, are calculated^169^.

36 $$\begin{aligned} \psi _{g,n,i}= & {} \sum _{\alpha =0}^{M} \widetilde{P}_\alpha (x_i) \psi _{g,n,i}^{[\alpha ]}; \ \ \ \widetilde{L}_{g,n,i} = \sum _{\alpha =0}^{M} \widetilde{P}_\alpha (x_i) \widetilde{L}_{g,n,i}^{[\alpha ]}, \end{aligned}$$

37 $$\begin{aligned} \widetilde{P}_\alpha (x_i)= & {} 2 \sqrt{\frac{2\alpha +1}{2\alpha -1}} \left[ \frac{2\alpha -1}{\alpha } \right] x_i \widetilde{P}_{\alpha -1}(x_i) - \sqrt{\frac{2\alpha +1}{2\alpha -3}} \left[ \frac{\alpha -1}{\alpha }\right] \widetilde{P}_{\alpha -2}(x_i), \ \text {with} \ \ \widetilde{P}_{0}(x_i) = 1; \widetilde{P}_1(x_i) = 2\sqrt{3} x_i \end{aligned}$$

## Results

The 1D, 2D and 3D high-order diamond difference schemes as well as the classical, linear and quadratic discontinuous Galerkin schemes are already implemented in the Dragon-5 solver^169–171^. This study aims to extend and detect any anomalies in the VHEE/UHEE multigroup formalism in the CEPXS-mode. Since VHEE and UHEE interact over much larger ranges than CONV-RT, larger spatial dimensions are necessary for complete beam attenuation. To accurately evaluate the Njoy-Dragon chain’s multigroup performance for VHEE/UHEE-RT, it is crucial to minimize interference with the BFP equation’s numerical resolution and avoid error compensation effects related to ray effects or the $S_N$ discretization of the 3D Boltzmann catastrophic kernel. Consequently, deterministic transport is limited to one spatial dimension. Pure numerical investigations for higher dimensions can be found elsewhere^171^. Njoy-Dragon computational schemes for VHEE/UHEE consist of $N_g=300$ groups, an $S_{16}$ Gauss-Legendre quadrature, a $P_{15}$ Legendre anisotropy, and $D_r\ge 100$ voxels. A convergence criterion of $10^{-5}$ was imposed on the internal iterations of the electron flux. VHEE medium self-polarization is corrected by the classical Sternheimer–Peierls density effect correction^172,173^. Geant-4, which has been qualified against experiments for VHEE and UHEE beams^66,68–72,74^, is the reference MC code used for validation purposes. A statistical uncertainty of $0.2\%$ or better is achieved in each voxel with the G4EmLivermore physics list constructor. Other legacy constructors, such as G4EmPenelope and GEmStandard^174^, were also used to verify the stability of Geant-4 response at VHEE and UHEE. The classical Lewis condensed-history (CH) theory^175^, implemented in Urbán’s algorithm ($\texttt {G4UrbanMscModel}$)^176^, is used below $\text{100 MeV}$ while the Goudsmit–Saunderson condensed-history theory^156,157^, implemented in the advanced Bagulya algorithm ($\texttt {G4GoudsmitSaundersonMscModel}$)^177^, is used for higher energies. The default G4MultipleScattering step size is used for MC soft collisions. Fluorescence and bremsstrahlung photons are produced in Geant-4 and Dragon-5 and immediately eliminated at the point of birth. Our aim is to retain purely electron transport, eliminating any coupling effect or dose deposition of a photonic nature. The resulting dose is therefore strictly electronic. This strategy ensures the BFP-MC comparison remains unaffected by this operation, owing to the equivalence of photonic elimination processes in both Geant-4 and Njoy-Dragon-5. Both codes use a $1~\text{keV}$ secondary production threshold and transport cutoff in compliance with the CEPXS-mode requirements in the ELECTR module.

### Radiation oncology benchmarks

**Figure 3:**
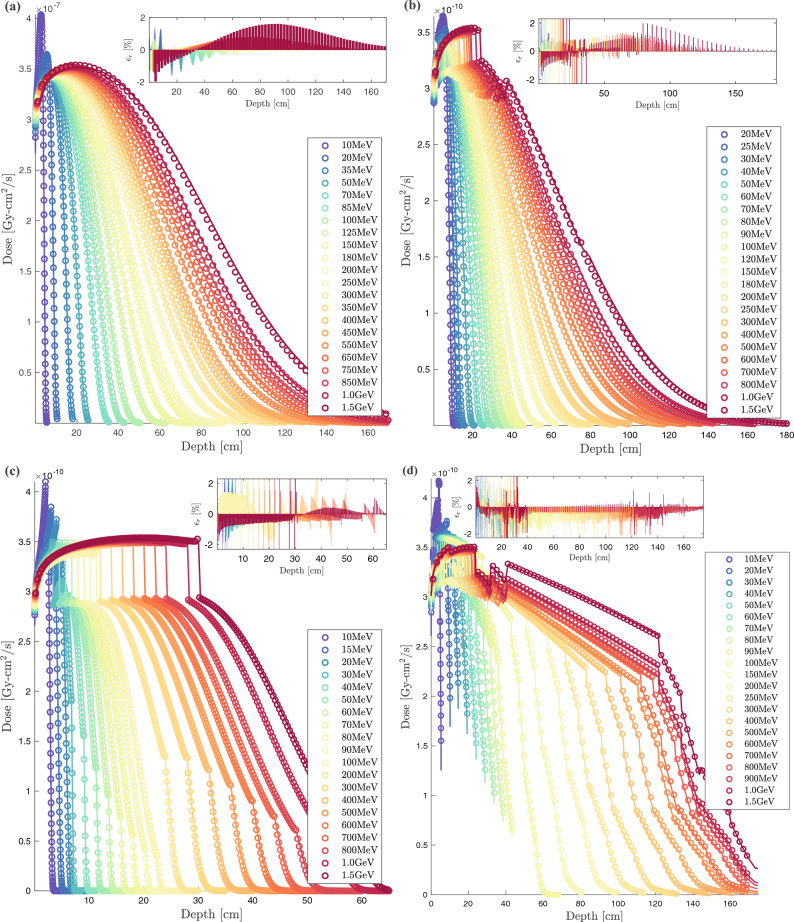
BFP depth-dose curves (solid lines) compared to MC (circles) for selected CONV, VHEE and UHEE unidirectional beams incident on: (**a**) Water benchmark; (**b**) Thorax benchmark: tissue [$13\%$], bone [$7\%$], lung [$22\%$] and tissue [$58\%$]; (**c**) IORT benchmark: tumor [$40\%$], aluminium [$40\%$], steel [$15\%$] and tissue [$5\%$] ; (**d**) high-heterogeneity patient-like benchmark: adipose [$5\%$], muscle [$7\%$], bone [$4\%$], muscle [$4\%$], lung [$41\%$], muscle [$6\%$], bone [$5\%$], adipose [$8\%$], bone [$7\%$], muscle [$6\%$] and adipose [$7\%$]. Insert shows BFP relative error with respect to MC.

**Figure 4:**
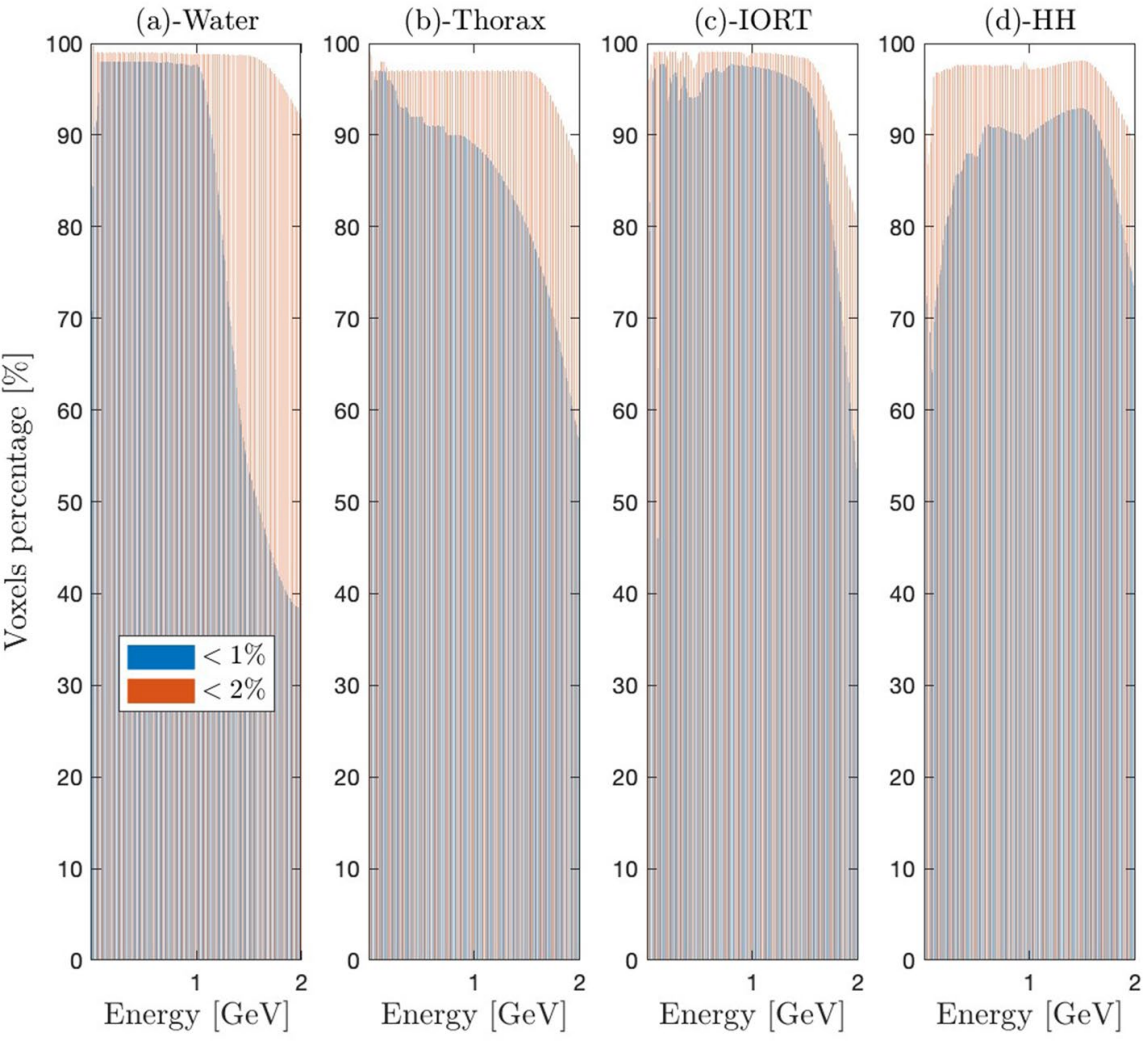
Percentage of BFP voxels with a relative deviation below $1\%$ ($\epsilon _1$) and $2\%$ ($\epsilon _2$) in reference to MC, for benchmarks: (**a**) Water; (**b**) Thorax; (**c**) IORT; (**d**) high-heterogeneity patient.

Figure 3a–d shows the dose profiles for four ascending transport complexity benchmarks^104^. The dimensions of each benchmark are determined iteratively based on the maximum range of the incident electron beam. The investigated beams range from $1~\text{MeV}$ to $6~\text{GeV}$, with a primary focus on the VHEE and UHEE domains. Insets display the relative deviations ($\epsilon _r$), expressed as a percentage, between the Njoy-Dragon chain and Geant-4. The error is computed using a fine piecewise cubic Hermite grid unification applied to both BFP and MC dose detectors. We have optimized the irradiation process for full beam attenuation to enhance electron transport challenges in this study, deliberately not conforming to typical clinical settings with smaller dimensions and, thus, less demanding buildup, backscattering, and attenuation scenarios. In Fig. 4a–d, the percentage of voxels within the Njoy-Dragon chain that satisfy a deviation criteria of less than $1\%$ ($\epsilon _1$) and $2\%$ ($\epsilon _2$) compared to Geant-4 is quantified for the same benchmarks. The $\epsilon _2$ criterion is deemed, within this context, the optimal instrument for assessing the performance of the BFP response. This premise is rooted in the standards set by the American Association of Physicists in Medicine (AAPM) for satisfactory dose prediction in CONV-RT^178,179^. The $\epsilon _1$ criterion, on the other hand, is seldom mentioned in existing literature. Its introduction in this study serves to underscore specific vulnerabilities. However, it should be perceived as an overly cautious exaggeration as the stated uncertainty on the cross-sections exceeds $1\%$.

All benchmark samples undergo irradiation with incremental energy levels as follows: increments of $1~\text{MeV}$ in the range of 1 to $20~\text{MeV}$, $5~\text{MeV}$ between 20 and $100~\text{MeV}$, and $20~\text{MeV}$ in the $100~\text{MeV}$ to $1~\text{GeV}$ interval. Beyond $1~\text{GeV}$, the increment increases to $500~\text{MeV}$. The selection of beams, as presented in Fig. 3a–d, has been made deliberately to enhance visual clarity and minimize overlap. However, the performance of the entire set of considered beams is comprehensively presented in Fig. 4a–d. We truncated the graphical performance display beyond $2~\text{GeV}$ as the extrapolation routines for the CEPXS-mode fail past this energy threshold.

Figures 3a and 4a depict the fundamental case of a spatially homogeneous water (W) slab. The high BFP-MC agreement seen in Fig. 3a leads to $99\%$ of the W-voxels satisfying the AAPM $\epsilon _2$ criterion (Fig. 4a) below $1.5~\text{GeV}$. Fig. 4a can also be compared to our previous result in CONV-RT^104^, wherein $100\%$ of voxels satisfied the $\epsilon _2$ criterion. The decrease in $\epsilon _2$ by 1 voxel beyond CONV-RT is attributed to our newly introduced deterministic computation scheme, which required a Legendre order of $P_{15}$ (in VHEE and UHEE) compared to $P_{8}$ and $P_{12}$ (in CONV). This necessitated a commensurate reduction in the $S_N$ order ($S_{16}$ in VHEE and UHEE vs. $S_{64}$ in CONV) and a five-fold decrease in spatial discretization. A Gauss-Legendre quadrature with $N = l + 1$ points is used here to ensure that the highly forward-peaked scattering is correctly integrated by the Boltzmann kernel. This forms what Morel defined as a Galerkin quadrature^180^, a discrete ordinates method that exactly integrates scattering represented by a delta function. When applying a more conservative $\epsilon _1$ criterion, the percentage of voxels decreases to 70.8–84.4% between 1 and $20~\text{MeV}$, 91.0–97.9% between 25 and $70~\text{MeV}$, 98.0–98.7% between 70 and $600~\text{MeV}$, and 97.9–97.7% between $601~\text{MeV}$ and $1.5~\text{GeV}$. Figure 4a displays a monotonic decrease in the conformity of BFP-MC above $1.5~\text{GeV}$. The adherence to $\epsilon _2$ ($\epsilon _1$) reaches $91.8\%$ ($38.3\%$) at $2~\text{GeV}$, subsequently decreases to $78.0\%$ ($18.7\%$) at $3~\text{GeV}$, and further diminishes to $53.3\%$ ($12.6\%$) at $5~\text{GeV}$. Optimization attempts beyond $2~\text{GeV}$ didn’t improve the downward trend in $\epsilon _2$. Thus, we deduce that ELECTR’s extrapolation routines are valid up to $1.5~\text{GeV}$, aligning with the interest limit in medical physics, negating the need for further development. The $\epsilon _1$ criterion emphasizes (Fig. 4a) the superior performance in VHEE and UHEE domains compared to the CONV range and beyond CONV. Figure 4a highlights that, for water, the $\epsilon _1$ criterion is affected during the $1.0-1.5~\text{GeV}$ transition without affecting the compliance with $\epsilon _2$. Taking all the W-voxels into account, the average absolute difference ($\bar{\epsilon }$) between BFP and MC is $0.19\%$, $0.23\%$, $0.30\%$, and $0.38\%$ at $100~\text{MeV}$, $300~\text{MeV}$, $500~\text{MeV}$, and $1~\text{GeV}$, respectively. It is noteworthy that this study remains fundamentally a proof-of-concept demonstrating the possibility of transcending the $100~\text{MeV}$ design limit of the CEPXS-mode. The qualification of Dragon-5 for a particular clinical routine will initiate iteration calculations on $N_g$, $P_l$, $S_N$ and $D_r$ deterministic parameters. Consequently, greater accuracy than those presented and tailored computation times could potentially be reported. Such parameter studies, as in nuclear reactor physics, are typically conducted and constructed on a case-by-case basis.

Figures 3b and 4b refer to the first level of heterogeneity with the thorax (T) benchmark. The thicknesses of the 4 slabs (muscle [$13\%$], bone [$7\%$], lung [$22\%$] and soft tissue [$58\%$]) are deduced from the maximum range of the incident beam’s energy. The BFP-MC T-dose profile agreement (Fig. 3b) is maintained regardless of the biological tissue’s nature, density, or thickness. The slight decrease in BFP-MC W-compliance concerning the AAPM $\epsilon _2$ criterion (Figs. 4b vs. 4a) can be attributed to the Geant-4 boundary crossing effects at the bone interface. This effect pertains solely to one voxel and appears only on one interface, the bone-lung interface. Its slight propagation in Fig. 4b is attributed to its spread to artificial voxels following a grid unification operation based on Hermite polynomials for BFP-MC comparison. As observed in Fig. 4b, the percentage of T-voxels satisfying the $\epsilon _2$ criterion reaches $99.2\%$ for energies between 1 and $20~\text{MeV}$. It then decreases and plateaus at around $97.0\%$ for energies up to $1.5~\text{GeV}$. Like the W-benchmark, the $\epsilon _1$ criterion in Fig. 4b emphasizes the BFP-MC compliance improvement at the $20~\text{MeV}$ threshold, i.e., the CONV-VHEE transition. The BFP-MC $\epsilon _1$ conformity increases from 65.8 to $93.6\%$ in CONV range, stabilizes around $97.0\%$ for energies between 25 and $200~\text{MeV}$, and then undergoes a monotonic decline from 97.0 to $80.1\%$ within the range of $200~\text{MeV}$ to $1.5~\text{GeV}$. We report, however, that $\bar{\epsilon }$ is $0.34\%$, $0.37\%$, $0.39\%$ and $0.44\%$, respectively, at $100~\text{MeV}$, $300~\text{MeV}$, $500~\text{MeV}$ and $1~\text{GeV}$.

Figures 3c and 4c introduce a second level of heterogeneity with the IORT Mobetron benchmark. The thicknesses of the tumor [$40\%$], aluminium [$40\%$], steel [$15\%$] and tissue [$5\%$] slabs are deduced in the same iterative methodology as for W- and T-slabs. Fig. 3c confirms that the IORT objective of a quasi-homogeneous dose within the tumor, followed by an immediate attenuation in the high-Z slabs inserted by the surgeon and finally a complete protection of healthy tissue is achieved for all beams. Fig. 4c illustrates that, for energies between 25 and $100~\text{MeV}$, 97 to $99\%$ of IORT-voxels meet the $\epsilon _2$ criterion. This is followed by a quasi-plateau around $99\%$ for energies between $100~\text{MeV}$ and $1.0~\text{GeV}$, ultimately reaching ${98.4}{\%}$ at $1.5~\text{GeV}$. A distinct drop is observed beyond $1.5~\text{GeV}$, reaching $90.2\%$ at $1.8~\text{GeV}$, $79.9\%$ at $2.0~\text{GeV}$, and descending to $62.0\%$ at $5.5~\text{GeV}$. The effect of the VHEEs insensitivity to the boundary crossing effects and heterogeneity level is better translated by the $\epsilon _1$ criterion. Figure 4c shows that the percentage of IORT-voxels meeting this criterion rises from 80.2 to $97.7\%$ as energy increases from $1~\text{MeV}$ to $100~\text{MeV}$. This percentage stabilizes around this value up to $1.0~\text{GeV}$, then slightly decreases to ${95.2}{\%}$ at $1.5~\text{GeV}$. Localized losses of accuracy, amounting to 1.2 and $1.8\%$ compared to the discussed performance, are observed at $300~\text{MeV}$ and $450~\text{MeV}$, respectively, as highlighted by both $\epsilon _1$ and $\epsilon _2$ in Fig. 4c. The superior BFP-MC compliance on the IORT-benchmark, when compared to the T-one (Fig. 4b vs. 4c), can be attributed to the absence of the thin osseous slab. Averaging over all IORT-voxels, $\bar{\epsilon }$ is $0.28\%$, $0.37\%$, $0.32\%$ and $0.27\%$, respectively, at $100~\text{MeV}$, $300~\text{MeV}$, $500~\text{MeV}$ and $1~\text{GeV}$.

Figure 3d displays the BFP and MC dose profiles for the highly heterogeneous (HH) patient-like benchmark. The heightened transport complexity arises from the number slabs and their variation in thickness, density, and biological nature. The composition includes adipose [$5\%$], muscle [$7\%$], bone [$4\%$], muscle [$4\%$], lung [$41\%$], muscle [$6\%$], bone [$5\%$], adipose [$8\%$], bone [$7\%$], muscle [$6\%$] and adipose [$7\%$]. The agreement between BFP and MC doses is maintained across all slabs, depths, buildups, densities, and beams. What was reported for the previous discussion regarding the CONV-VHEE transition remains true for HH-benchmark. Figure 4d indicates that for VHEE below $100~\text{MeV}$, the $\epsilon _2$ criterion is met by 93.7 to $96.8\%$ of the HH-voxels. The fulfillment rate slightly rises to 96.8–97.7% for energies from $100~\text{MeV}$ to $1~\text{GeV}$, then further increases and stabilizes around $98.1\%$ up to $1.5~\text{GeV}$. When the more stringent $\epsilon _1$ criterion is applied, these percentages drop to $70.4\%$, 70.4–91.1%, and $92.9\%$, respectively. Restricting consideration to the $\epsilon _2$ criterion, a slightly improved BFP-MC compliance can be readily observed in Fig. 4 during the transition from the T to HH-benchmark. This is due to the compensatory effects of backscattering from adjacent slabs, given the tripling of the total number of slabs during the T-HH transition. However, the $\epsilon _1$ criterion indicates the opposite. This can be attributed to the substantial difficulty in achieving compliance below $1\%$ within tissues when transitioning from 4 slabs to 11 slabs. When averaged across all HH-voxels, $\bar{\epsilon }$ is $0.55\%$, $0.61\%$, $0.68\%$ and $0.70\%$, respectively, at $100~\text{MeV}$, $300~\text{MeV}$, $500~\text{MeV}$ and $1~\text{GeV}$. All violations of the $\epsilon _2$ criterion observed in the insets of Figs. 3a–d are attributed to specific interface issues on the Geant-4 side.

The hypothesis of a BFP-MC dose equivalence fails to be rejected by the Kolmogrov-Smirnov goodness-of-fit test at a significance level of $\sim0.02$ below $1.5~\text{GeV}$ for all benchmarks, indicating a high level of BFP-MC compliance. The Dragon-5 CPU time, which depends solely on deterministic parameters ($N_g$, $P_l$, $S_N$, and $D_r$), remains consistent at 5 seconds across all beams. In contrast, the Geant-4 CPU time increases with the beam energy, with estimated times of 10.4 days, 24.9 days, and 36.36 days at $100~\text{MeV}$, $500~\text{MeV}$, and $1~\text{GeV}$, respectively. These comparisons were conducted on an Intel Xenon E5-2683 v4 CPU.

### High-Z benchmarks

**Figure 5:**
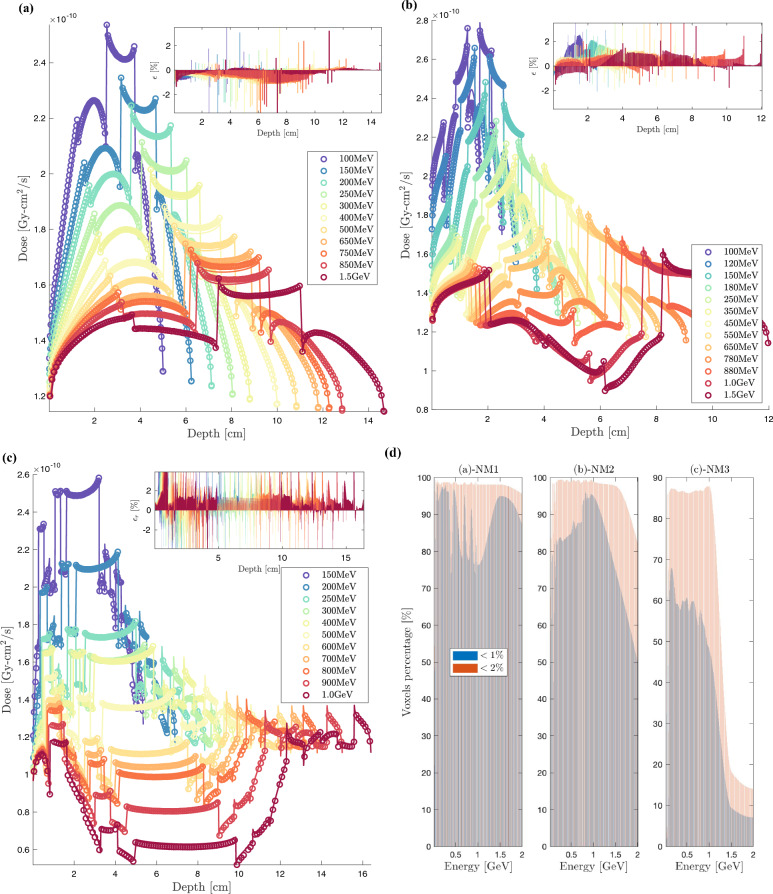
BFP depth-dose curves (solid lines) compared to MC (circles) for selected VHEE and UHEE unidirectional beams incident on: (**a**) NM1-benchmark: $_{26}$Fe [$25\%$], $_{33}$As [$25\%$], $_{6}$C [$25\%$] and $_{40}$Zr [$25\%$]; (**b**) NM2-benchmark: $_{14}$Si [$17\%$], $_{42}$Mo [$17\%$], $_{24}$Cr [$17\%$], $_{87}$Fr [$17\%$], $_{12}$Mg [$17\%$] and $_{29}$Cu [$15\%$]; (**c**) NM3-benchmark: $_{79}$Au [$10\%$], $_{16}$S [$10\%$], $_{30}$Zn [$10\%$], $_{50}$Sn [$10\%$], $_{11}$Na [$10\%$], $_{34}$Se [$10\%$], $_{19}$K [$30\%$], $_{62}$Sm [$10\%$], $_{23}$V [$10\%$], $_{46}$Pd [$10\%$], $_{5}$B [$10\%$], $_{39}$Y [$10\%$], $_{49}$In [$10\%$], $_{70}$Yb [$10\%$], and $_{22}$Ti [$10\%$]. (**d**) Percentage of BFP voxels with a relative deviation below $1\%$ ($\epsilon _1$) and $2\%$ ($\epsilon _2$) in reference to MC. Insert shows BFP relative error with respect to MC.

We propose to amplify the complexity of transport by incorporating slabs of high heterogeneity, high density and high atomic number. Simultaneously, we enhance unidirectional beam incidences by initiating irradiation from both sides of the benchmarks. Figure 5a presents the BFP dose vs. MC for the non-medical benchmark NM1: $_{26}$Fe [$25\%$], $_{33}$As [$25\%$], $_{6}$C [$25\%$] and $_{40}$Zr [$25\%$]. Figure 5d illustrates that the percentage of NM1-voxels satisfying the $\epsilon _2$ criterion fluctuates around $98.8\%$ up to ${1.5}\quad {\textrm{GeV}}$. Beyond this, a continuous decline is observed, reaching $95.6\%$, $79.7\%$, $73.1\%$, $69.3\%$, and $68.7\%$ at $2~\text{GeV}$, $3~\text{GeV}$, $4~\text{GeV}$, $5~\text{GeV}$, and $6~\text{GeV}$, respectively. The $\epsilon _1$ criterion demonstrates a significant loss of accuracy for certain well-distinguished energies, while for other energies, the criterion remains satisfied at 95.1–97.5%. Figure 5a reveals that significant BFP-MC discrepancies are largely localized within the carbon slab for specific beams, most notably at $300~\text{MeV}$, $400~\text{MeV}$, and $850~\text{MeV}$, but this pattern is not universal. Our impending meta-analysis will underscore that carbon does not pose any significant challenge for the BFP solution within VHEE and UHEE domains. However, the origins of these discrepancies within specific beams remain unclear. When averaged across all NM1-voxels, the mean BFP-MC discrepancy $\bar{\epsilon }$ stands at $0.34\%$, $0.44\%$, $0.51\%$, and $0.65\%$ for $100~\text{MeV}$, $300~\text{MeV}$, $500~\text{MeV}$, and $1~\text{GeV}$, respectively.

Figure 5b presents dose profiles for the NM2 benchmark: $_{14}$Si [$17\%$], $_{42}$Mo [$17\%$], $_{24}$Cr [$17\%$], $_{87}$Fr [$17\%$], $_{12}$Mg [$17\%$] and $_{29}$Cu [$15\%$]. As observed with the NM1 case, NM2 displays similar trends with subtle differences in conformity. In Fig. 5d, the BFP-MC conformity for the $\epsilon _2$ criterion oscillates between 98.3 and $99.4\%$ within the $100~\text{MeV}$–$1.5~\text{GeV}$ energy range. Above this range, we observe a consistent decline. Below it, the BFP-MC conformity to the mentioned criterion climbs from 86.1 to $98.3\%$. With the $\epsilon _1$ criterion, we can distinguish the clear pattern of BFP-MC convergence that escalates with increasing beam energy: BFP-MC conformity rises from $77.2\%$ at $61~\text{MeV}$ to $95.0\%$ at $1~\text{GeV}$, beyond which the conformity starts to diminish. Transitioning from NM1 to NM2 brings increased complexity in transport, seen in the expanded number of slabs and the more intricate irradiated materials. This increase in complexity correlates with a decrease in conformity for the stringent $\epsilon _1$ criterion. The diverging NM2-voxels are located in the fourth slab of francium, which possesses the highest atomic number among all NM2 slabs. The upcoming meta-analysis will show that francium categorically exhibits a BFP-MC deviation above $2\%$ at the point of maximum energy deposition for all VHEE and UHEE beams. Taking an average over all the NM2 voxels, $\bar{\epsilon }$ measures $0.62\%$, $0.52\%$, $0.60\%$, and $0.43\%$ at $100~\text{MeV}$, $300~\text{MeV}$, $500~\text{MeV}$, and $1~\text{GeV}$, respectively.

Figure 5c relates to the benchmark with the highest level of complexity, NM3, composed of 15 slabs, from left to right: $_{79}$Au [$10\%$], $_{16}$S [$10\%$], $_{30}$Zn [$10\%$], $_{50}$Sn [$10\%$], $_{11}$Na [$10\%$], $_{34}$Se [$10\%$], $_{19}$K [$30\%$], $_{62}$Sm [$10\%$], $_{23}$V [$10\%$], $_{46}$Pd [$10\%$], $_{5}$B [$10\%$], $_{39}$Y [$10\%$], $_{49}$In [$10\%$], $_{70}$Yb [$10\%$], and $_{22}$Ti [$10\%$]. The complexity of transport is illustrated by compliance with both $\epsilon _1$ and $\epsilon _2$ criteria, as depicted in Fig. 5d. Between $100~\text{MeV}$ and $1~\text{GeV}$, compliance with the $\epsilon _2$ criterion fluctuates between 87.2 and $87.9\%$, but it drops significantly to $20.5\%$ at $1.5~\text{GeV}$. Conversely, it increases from 80.4 to $87.2\%$ as energy transitions from 45 to $99~\text{MeV}$. It was previously demonstrated that $1.5~\text{GeV}$ was the maximum limit to which the CEPXS-mode could be extended from its design limit of $100~\text{MeV}$. This was the case for all previous benchmarks, except for NM3. For the latter, the compliance with the $\epsilon _1$ criterion is the lowest observed, with a maximum of $68.0\%$ at $150~\text{MeV}$ (Fig. 5d). Notably, voxels showing significant deviations are predominantly located in the seventh slab of phosphorus, the largest slab of the benchmark. Intriguingly, just as observed in NM1, phosphorus does not pose a transport challenge. Hence, the observed deviation potentially implicates the type of geometric encapsulation of the slab. This scenario underscores the NM3-benchmark’s need for meticulous optimization of deterministic computation schemes, emphasizing spatial discretization, quadratures, and anisotropy order, to enhance BFP-MC compliance. Averaged over all NM3 voxels, $\bar{\epsilon }$ are $1.35\%$, $1.38\%$, $1.35\%$, and $1.36\%$ at $100~\text{MeV}$, $300~\text{MeV}$, $500~\text{MeV}$, and $1~\text{GeV}$, respectively.

### VHEE and UHEE meta-analysis

**Figure 6:**
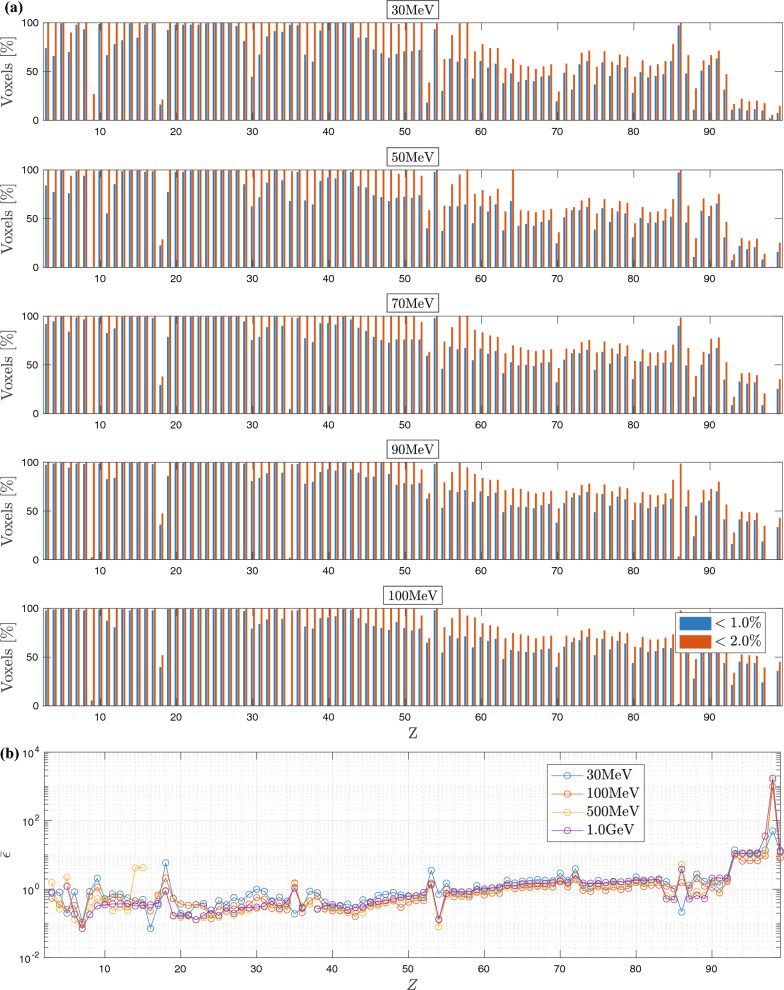
(**a**) Homogeneous atomic slabs’ voxels percentage satisfying a $1\%$ and $2\%$ BFP-MC relative dose difference vs. *Z* for selected VHEE and UHEE beams; (**b**) BFP-MC mean relative error vs. *Z*. Monte Carlo convergences are obtained for a $0.2\%$ mean standard deviation.

**Figure 7:**
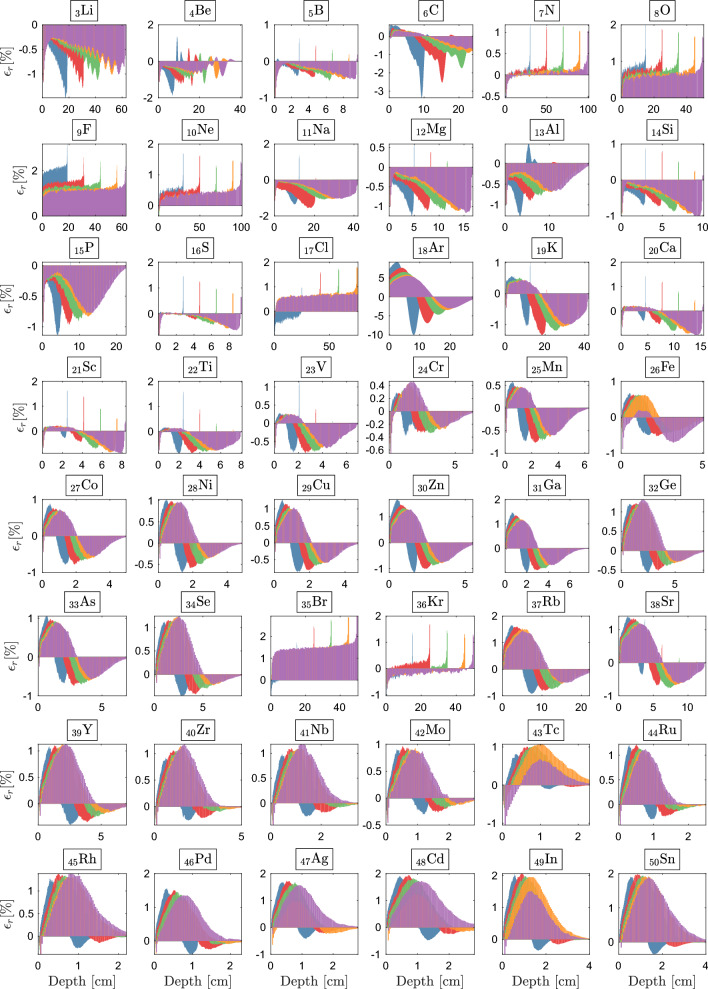
BFP-MC relative differences in energy deposition profiles vs. depth for selected $30~\text{MeV}$, $50~\text{MeV}$, $70~\text{MeV}$, $90~\text{MeV}$ and $100~\text{MeV}$ from $Z=35$ to $Z=50$. Irradiated slab dimensions correspond to beam range. Achieved Monte Carlo convergence with $0.2\%$ mean standard deviation.

**Figure 8:**
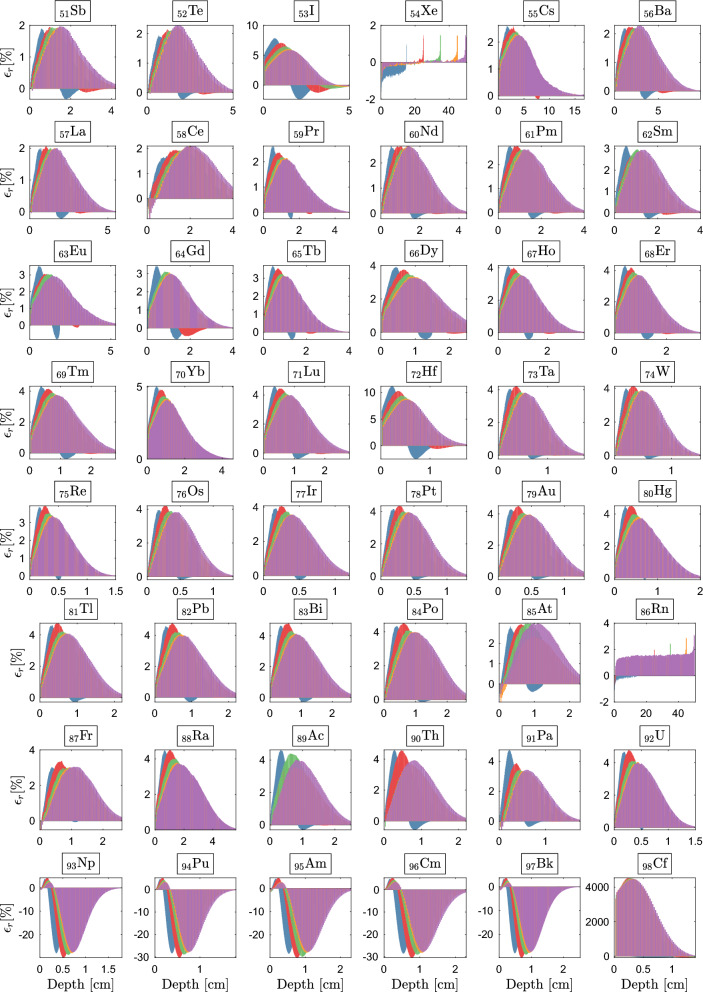
BFP-MC relative differences in energy deposition profiles vs. depth for selected $30~\text{MeV}$, $50~\text{MeV}$, $70~\text{MeV}$, $90~\text{MeV}$ and $100~\text{MeV}$ from $Z=51$ to $Z=98$. Irradiated slab dimensions correspond to beam range. Achieved Monte Carlo convergence with $0.2\%$ mean standard deviation.

Here, we probe the boundaries of the extended CEPXS-mode in ELECTR at VHEE and UHEE, scrutinizing the Class concept’s validity from CONV-RT^104^. We irradiate the entire periodic table, from hydrogen to einsteinium, with unidirectional beams under VHEE and UHEE conditions. For each irradiation, an iterative procedure autonomously determines the slab dimensions in Geant-4 and Dragon-5, taking into account the beam energy, irradiated material, and corresponding stopping power. Figure 6a shows the behaviour of criteria $\epsilon _1$ and $\epsilon _2$ as a function of *Z*, while Fig. 6b highlights the same behaviour for $\bar{\epsilon }$. Figures 7–8 illustrate the spatial distribution of $\epsilon _r$ for most elements.

Tracking the behavior of the maximum relative error across Figs. 7–8, we observe the compliance of the BFP-MC deviation with respect to the $\epsilon _2$ criterion. We note that the maximum deviation is often located at the benchmark’s *hottest point*, i.e., the point of maximum energy deposit ($d_{\text {max}}$). Except for a few exceptions, Figs. 7–8 show that the $\epsilon _2$ criterion is respected at VHEE and UHEE from $Z=3$ (lithium) to $Z=58$ (cerium). This consistency constitutes what we define as the first response Class, $\mathscr {C}_{1}$, which aligns with the criteria established for CONV-RT^104^. Illustratively, for $\mathscr {C}_{1}$-elements, and within an energy range of ${30} \quad{\textrm{MeV}}$ to ${1}\quad {\textrm{GeV}}$, the combined maximum deviation for all voxels does not exceed $0.6\%$ for $_{5}$B, $0.8\%$ for $_{14}$Si, $1.6\%$ for $_{21}$Sc, $1.2\%$ for $_{29}$Cu, $1.2\%$ for $_{41}$Nb, $1.9\%$ for $_{48}$Cd and $1.98\%$ for $_{57}$La.

From $Z=59$ (praseodymium) to $Z=92$ (uranium), we observe the formation of a second response Class, $\mathscr {C}_{2}$. Three distinct characteristics define this Class: (1) the systematic appearance of a deviation above $2\%$ at $d_{\text {max}}$; (2) the persistence of this behavior across all $\mathscr {C}_{2}$-elements and all beams studied; and (3) a gradual yet consistent increase and lateral expansion in this deviation with increasing *Z*.

As an illustration, at $d_{\text {max}}$, $\epsilon _{\text {max}}$ reaches $2.6\%$ for $_{60}$Nd, $3.7\%$ for $_{65}$Tb, $4.1\%$ for $_{74}$W, $3.0\%$ for $_{85}$At, $4.6\%$ for $_{89}$Ac, and $4.8\%$ for $_{92}$U.

A shift towards a third response Class ($\mathscr {C}_{3}$) is observed beginning at $Z=93$ (neptunium) and continuing up to $Z=99$ (einsteinium). In $\mathscr {C}_{3}$, the BFP-MC deviations initially quadruple, then increase tenfold as *Z* increases. $\epsilon _{\text {max}}$ reaches $29.9\%$, $30.3\%$, $29.8\%$, $30.4\%$, $29.8\%$, $4419.6\%$ and $30.6\%$, respectively for $_{93}$Np, $_{94}$Pu, $_{95}$Am, $_{96}$Cm, $_{97}$Bk, $_{98}$Cf and $_{99}$Es. Another unique aspect within $\mathscr {C}_{3}$ is that a substantial majority of the voxels become implicated in the violation of the $\epsilon _2$ criterion (Fig. 8). We therefore conclude that, under VHEE and UHEE conditions, the extended CEPXS-mode is operational for $\mathscr {C}_{1}$, rejected for $\mathscr {C}_{2}$ and entirely ineffective for $\mathscr {C}_{3}$.

The deviation from the anticipated behavior of $\mathscr {C}_{1}$ is manifested across four distinct categories of elements: three noble gases—helium ($_{2}$He), neon ($_{10}$Ne), and argon ($_{18}$Ar); three halogens—fluorine ($_{9}$F), bromine ($_{35}$Br), and iodine ($_{53}$I); two gases, namely hydrogen ($_{1}$H) and oxygen ($_{8}$O); and two metals—lithium ($_{3}$Li) and boron ($_{5}$B). The transitions between the response Classes—from $\mathscr {C}_{1}$ to $\mathscr {C}_{2}$, and subsequently the fall into $\mathscr {C}_{3}$—are distinctly depicted across the full VHEE and UHEE range, as showcased by the $\epsilon _2$ criterion in Fig. 6a. Additionally, the $\epsilon _1$ criterion (Fig. 6a) elucidates how the convergence of BFP-MC strengthens with increasing beam energy within the same Class. Remarkably, a significant decrease in both $\epsilon _1$ and $\epsilon _2$ values within the same Class (Fig. 6a) facilitates the identification of the previously mentioned exceptions to our classification methodology. In terms of $\bar{\epsilon }$, Fig. 6b shows that the $\mathscr {C}_{1}$-elements demonstrate a consistent $\bar{\epsilon }$ below $1.0\%$, clearly distinguishing them. The transition to $\mathscr {C}_{2}$ is marked by a noticeable shift to $\bar{\epsilon }=1.0\%$, which then gradually settles around $\bar{\epsilon }=1.98\%$. The move to $\mathscr {C}_{3}$ is even more dramatic, as depicted in Fig. 6b. This transition initiates an abrupt increase from $\bar{\epsilon }=2.00\%$ for $_{92}$U to $\bar{\epsilon }=10.73\%$ for $_{93}$Np, yet it doesn’t inhibit the recording of an extraordinary average deviation of ${1719.88}{\%}$ for $_{98}$Cf. Ultimately, the irregularities observed in Fig. 6a of an atom relative to its neighbors are intrinsic to the CEPXS mode. Our ongoing study indicates that the ENDF mode in ELECTR fully resolves these irregularities and failures beyond Z=50.

## Study limits

*Transverse transport:* A deliberate decision was taken to validate the proposed extension through deterministic transport, rather than contrasting multigroup cross sections. Therefore, there’s a skipped layer which involves extensive comparison of multigroup cross-sections–namely catastrophic transfer matrices (Eqs. 3–22), total catastrophic cross-sections (Eq. 23), soft stopping powers (Eqs. 24–25), and energy deposition cross-sections (Eq. 26)-with their credited, certified, or evaluated counterparts. It was then essential to pinpoint the pure influence of multigroup atomic data on electron transport. One approach to accomplish this is by restricting transport to one dimension, thereby eliminating any external interference or additional factors that might affect the direct observation of the multigroup cross sections’ impact on BFP transport. BFP 1D resolution is known to be exact. In contrast, 2D and 3D transverse transport intervene with ray effects and SN catastrophic kernel discretization effects. Hence, it is imperative that 2D and 3D deterministic numerical challenges don’t skrew the relative BFP-MC discrepancy, whether by amplifying it through additive factors or lessening it with compensatory ones. Transverse electron transport interferes our main objective: to clearly identify and separate the effects of multigroup cross-sections on electron transport. Dragon-5 transverse transport capacity can be found elsewhere (e.g., Fig.11, p.12 of Ref.^169^). Moreover, since the cross sections are spatially anisotropic, the transverse transport capabilities are not affected by the cross sections.

*Photon transport:* The BFP dose calculation is predetermined by the energy deposition cross section (Eq. 26). This quantity is (1) complex; (2) deterministic; and (3) has no equivalent in MC paradigm. The successes and failures reported in this study are more a reflection of the quality of this specific cross section. Therefore, to validate “pure electron” transport, it is important to maintain a pure electron energy deposition cross section. The production of fluorescence and bremsstrahlung spectra is already implemented in ELECTR. A coupled photon-electron calculation in Dragon-5 is straightforward. However, from the Njoy perspective, it requires the coupling of two multigroup nuclear data modules; GAMINR for photons and ELECTR for electrons. Such coupling is essential to address the photons that emerge from electron transport—including fluorescence and bremsstrahlung—as well as the electrons produced by photon interactions, namely the photoelectric effect, Compton scattering, pair production, and triplet production. This coupling goes beyond the scope of this study and has not yet been performed.

*Crossing boundary effect:* A portion of the BFP-MC discrepancy is due to Geant-4’s inability to accurately predict the dose at the interface point. In contrast, Dragon-5’s predictions at the interface are highly realistic. This topic has been thoroughly addressed in our previous paper (see Figs. 3 and 4 and p. 12-13 of Ref.^104^). Two methods can be applied to correct Geant-4’s predictions: (1) by forcing the electron step size not to exceed $25\%$ of the voxel’s thickness; or (2) by optimizing the control constants of the G4UrbanMscModel condensed history (CH) algorithm. Such modifications to the MC computational scheme are recognized as extremely time-consuming. Therefore, they are not practical for VHEE and UHEE beams, which are already very demanding in terms of CPU time.

## Conclusion

Very-High Energy Electron (VHEE) and Ultra-High Energy Electron (UHEE) treatments are currently in their advanced preclinical testing stage. The Monte Carlo (MC) solution currently serves as the only operational transport support for experimental trials and forthcoming clinical deployments. However, its computational intensity and time consumption make it less suitable for real-time, or *on-the-fly*, clinical applications. In this paper, we propose a potential alternative by extending the Boltzmann-Fokker-Planck (BFP) chain, Njoy-Dragon, for VHEE and UHEE beams. The energy range examined covers $1~\text{MeV}$ to $6~\text{GeV}$. We validated our multigroup solution in comparison with Geant-4, which has been previously qualified against experiments for VHEE and UHEE conditions. The validation process was conducted along three fronts: the first involves typical radio-oncology benchmarks for increasingly complex scenarios, the second focuses on benchmarks characterized by high levels of heterogeneity and intricate atomic structure, and the third involves irradiation of the entire periodic table.

By comparing the dose in each voxel, we found that $99\%$ of water voxels exhibited a BFP-MC deviation below $2\%$ under $1.5~\text{GeV}$. Similarly, applying the same criterion, $97\%$ and $99\%$ of thorax and breast intra-operative voxels, respectively, met this criterion above $50~\text{MeV}$. In the thorax, we observed a loss of 1 voxel of accuracy, attributable to the bone-lung interface. However, this issue was not further investigated on the Geant-4 side. For a heterogeneity level consisting of 11 slabs—including muscle, adipose, lung, and bone, 97–98% of the voxels displayed a BFP-MC deviation below $2\%$ between $60~\text{MeV}$ and $1.5~\text{GeV}$. These findings suggest that the accuracy gain following the mentioned energy thresholds is likely due to the insensitivity of VHEE/UHEE to the level of heterogeneity. While BFP-MC conformity at $1\%$ was analyzed, it is crucial to note that this criterion is lower than the uncertainties on cross-sections. Applying this criterion, we noted a decrease in voxel complicity: a reduction of $1\%$ for water voxels, 1–8% for thorax, 2–12% for Mobetron, and $7\%$ for the highly heterogeneous benchmark. The average BFP-MC discrepancy remained around $0.3\%$ and $0.4\%$ for water, thorax, and Mobetron at $300~\text{MeV}$ and $1~\text{GeV}$. However, it slightly increased to $0.6\%$ and $0.7\%$ for the highly heterogeneous benchmark.

For the second study, we involve, permute, and assemble high and medium *Z* materials while gradually increasing the level of heterogeneity and multiplying the incident beams. Below $1.5~\text{GeV}$, we achieved a minimum of $98.2\%$ BFP-MC compliance to the $2\%$ criterion in the Fe-As-C-Zr assembly. Meanwhile, for the Si-Mo-Cr-Fr-Mg-Cu assembly, compliance to the same criterion ranged between $97.8\%$ and $99.4\%$ within the $[75~\text{MeV},1.5~\text{GeV}]$ interval. However, in the more complex Au-S-Zn-Sn-Na-Se-K-Sm-V-Pd-B-Y-In-Yb-Ti assembly, BFP-MC compliance failed to exceed $87.9\%$ of all involved voxels and beams. This suggests that increased heterogeneity leads to a loss of accuracy at lower energy, particularly for the highly stringent $1\%$ criterion. The average BFP-MC discrepancy for assemblies 1 to 3 was found to be $0.44\%$ ($0.65\%$), $0.52\%$ ($0.43\%$), and $1.38\%$ ($1.36\%$) at $300~\text{MeV}$ ($1~\text{GeV}$), respectively. VHEE and UHEE irradiation of the entire periodic table has identified three classes of response. For elements with atomic numbers lower than praseodymium ($Z=59$), a BFP-MC discrepancy below $2\%$ was consistently achieved for all voxels within the $1~\text{MeV}$ to $1.5~\text{GeV}$ range. For elements from praseodymium to uranium, a systematic BFP-MC deviation slightly above $2\%$ was observed at the point of maximum energy deposit. This deviation not only persisted but also increased and widened laterally as *Z* increased. A third category was identified between neptunium and einsteinium, where the discrepancies were at least four times as large. However, we noted some exceptions to our classification, such as hydrogen, the noble gases helium and argon, and the halogens bromine and iodine.

The present study avoided any interference or counterbalancing effect between errors related to the multigroup formalism, ray effect, or the $S_{N}$ discretization of the Boltzmann kernel. For this reason, deterministic transport was limited to one dimension. The optimization of the deterministic computational scheme must be adapted to clinical routine. Even better results can be reported in the future with a refinement of the computational scheme (spatial discretization, Legendre order, $S_{N}$ quadrature and number of groups) and with the Evaluated Nuclear Data File (ENDF) mode in Njoy.

## Data Availability

The Njoy system is freely available under the Berkeley Software Distribution (BSD) 3-clause license. Similarly, the Boltzmann-Fokker-Planck solver, Dragon-5, is licensed under the GNU Lesser General Public License. All computational schemes and MATXS-formatted libraries can be obtained from the corresponding author (AN) upon reasonable request. The ENDF-mode in ELECTR will be freely accessible under the BSD 3-clause license by the end of 2024.
